# Identification and rejection of pile-up jets at high pseudorapidity with the ATLAS detector

**DOI:** 10.1140/epjc/s10052-017-5081-5

**Published:** 2017-09-02

**Authors:** M. Aaboud, G. Aad, B. Abbott, J. Abdallah, O. Abdinov, B. Abeloos, S. H. Abidi, O. S. AbouZeid, N. L. Abraham, H. Abramowicz, H. Abreu, R. Abreu, Y. Abulaiti, B. S. Acharya, S. Adachi, L. Adamczyk, J. Adelman, M. Adersberger, T. Adye, A. A. Affolder, T. Agatonovic-Jovin, C. Agheorghiesei, J. A. Aguilar-Saavedra, S. P. Ahlen, F. Ahmadov, G. Aielli, S. Akatsuka, H. Akerstedt, T. P. A. Åkesson, E. Akilli, A. V. Akimov, G. L. Alberghi, J. Albert, P. Albicocco, M. J. Alconada Verzini, M. Aleksa, I. N. Aleksandrov, C. Alexa, G. Alexander, T. Alexopoulos, M. Alhroob, B. Ali, M. Aliev, G. Alimonti, J. Alison, S. P. Alkire, B. M. M. Allbrooke, B. W. Allen, P. P. Allport, A. Aloisio, A. Alonso, F. Alonso, C. Alpigiani, A. A. Alshehri, M. Alstaty, B. Alvarez Gonzalez, D. Álvarez Piqueras, M. G. Alviggi, B. T. Amadio, Y. Amaral Coutinho, C. Amelung, D. Amidei, S. P. Amor Dos Santos, A. Amorim, S. Amoroso, G. Amundsen, C. Anastopoulos, L. S. Ancu, N. Andari, T. Andeen, C. F. Anders, J. K. Anders, K. J. Anderson, A. Andreazza, V. Andrei, S. Angelidakis, I. Angelozzi, A. Angerami, A. V. Anisenkov, N. Anjos, A. Annovi, C. Antel, M. Antonelli, A. Antonov, D. J. Antrim, F. Anulli, M. Aoki, L. Aperio Bella, G. Arabidze, Y. Arai, J. P. Araque, V. Araujo Ferraz, A. T. H. Arce, R. E. Ardell, F. A. Arduh, J.-F. Arguin, S. Argyropoulos, M. Arik, A. J. Armbruster, L. J. Armitage, O. Arnaez, H. Arnold, M. Arratia, O. Arslan, A. Artamonov, G. Artoni, S. Artz, S. Asai, N. Asbah, A. Ashkenazi, L. Asquith, K. Assamagan, R. Astalos, M. Atkinson, N. B. Atlay, K. Augsten, G. Avolio, B. Axen, M. K. Ayoub, G. Azuelos, A. E. Baas, M. J. Baca, H. Bachacou, K. Bachas, M. Backes, M. Backhaus, P. Bagnaia, H. Bahrasemani, J. T. Baines, M. Bajic, O. K. Baker, E. M. Baldin, P. Balek, F. Balli, W. K. Balunas, E. Banas, Sw. Banerjee, A. A. E. Bannoura, L. Barak, E. L. Barberio, D. Barberis, M. Barbero, T. Barillari, M.-S. Barisits, T. Barklow, N. Barlow, S. L. Barnes, B. M. Barnett, R. M. Barnett, Z. Barnovska-Blenessy, A. Baroncelli, G. Barone, A. J. Barr, L. Barranco Navarro, F. Barreiro, J. Barreiro Guimarães da Costa, R. Bartoldus, A. E. Barton, P. Bartos, A. Basalaev, A. Bassalat, R. L. Bates, S. J. Batista, J. R. Batley, M. Battaglia, M. Bauce, F. Bauer, H. S. Bawa, J. B. Beacham, M. D. Beattie, T. Beau, P. H. Beauchemin, P. Bechtle, H. P. Beck, K. Becker, M. Becker, M. Beckingham, C. Becot, A. J. Beddall, A. Beddall, V. A. Bednyakov, M. Bedognetti, C. P. Bee, T. A. Beermann, M. Begalli, M. Begel, J. K. Behr, A. S. Bell, G. Bella, L. Bellagamba, A. Bellerive, M. Bellomo, K. Belotskiy, O. Beltramello, N. L. Belyaev, O. Benary, D. Benchekroun, M. Bender, K. Bendtz, N. Benekos, Y. Benhammou, E. Benhar Noccioli, J. Benitez, D. P. Benjamin, M. Benoit, J. R. Bensinger, S. Bentvelsen, L. Beresford, M. Beretta, D. Berge, E. Bergeaas Kuutmann, N. Berger, J. Beringer, S. Berlendis, N. R. Bernard, G. Bernardi, C. Bernius, F. U. Bernlochner, T. Berry, P. Berta, C. Bertella, G. Bertoli, F. Bertolucci, I. A. Bertram, C. Bertsche, D. Bertsche, G. J. Besjes, O. Bessidskaia Bylund, M. Bessner, N. Besson, C. Betancourt, A. Bethani, S. Bethke, A. J. Bevan, J. Beyer, R. M. Bianchi, O. Biebel, D. Biedermann, R. Bielski, N. V. Biesuz, M. Biglietti, J. Bilbao De Mendizabal, T. R. V. Billoud, H. Bilokon, M. Bindi, A. Bingul, C. Bini, S. Biondi, T. Bisanz, C. Bittrich, D. M. Bjergaard, C. W. Black, J. E. Black, K. M. Black, R. E. Blair, T. Blazek, I. Bloch, C. Blocker, A. Blue, W. Blum, U. Blumenschein, S. Blunier, G. J. Bobbink, V. S. Bobrovnikov, S. S. Bocchetta, A. Bocci, C. Bock, M. Boehler, D. Boerner, D. Bogavac, A. G. Bogdanchikov, C. Bohm, V. Boisvert, P. Bokan, T. Bold, A. S. Boldyrev, A. E. Bolz, M. Bomben, M. Bona, M. Boonekamp, A. Borisov, G. Borissov, J. Bortfeldt, D. Bortoletto, V. Bortolotto, D. Boscherini, M. Bosman, J. D. Bossio Sola, J. Boudreau, J. Bouffard, E. V. Bouhova-Thacker, D. Boumediene, C. Bourdarios, S. K. Boutle, A. Boveia, J. Boyd, I. R. Boyko, J. Bracinik, A. Brandt, G. Brandt, O. Brandt, U. Bratzler, B. Brau, J. E. Brau, W. D. Breaden Madden, K. Brendlinger, A. J. Brennan, L. Brenner, R. Brenner, S. Bressler, D. L. Briglin, T. M. Bristow, D. Britton, D. Britzger, F. M. Brochu, I. Brock, R. Brock, G. Brooijmans, T. Brooks, W. K. Brooks, J. Brosamer, E. Brost, J. H Broughton, P. A. Bruckman de Renstrom, D. Bruncko, A. Bruni, G. Bruni, L. S. Bruni, BH Brunt, M. Bruschi, N. Bruscino, P. Bryant, L. Bryngemark, T. Buanes, Q. Buat, P. Buchholz, A. G. Buckley, I. A. Budagov, F. Buehrer, M. K. Bugge, O. Bulekov, D. Bullock, T. J. Burch, H. Burckhart, S. Burdin, C. D. Burgard, A. M. Burger, B. Burghgrave, K. Burka, S. Burke, I. Burmeister, J. T. P. Burr, E. Busato, D. Büscher, V. Büscher, P. Bussey, J. M. Butler, C. M. Buttar, J. M. Butterworth, P. Butti, W. Buttinger, A. Buzatu, A. R. Buzykaev, S. Cabrera Urbán, D. Caforio, V. M. Cairo, O. Cakir, N. Calace, P. Calafiura, A. Calandri, G. Calderini, P. Calfayan, G. Callea, L. P. Caloba, S. Calvente Lopez, D. Calvet, S. Calvet, T. P. Calvet, R. Camacho Toro, S. Camarda, P. Camarri, D. Cameron, R. Caminal Armadans, C. Camincher, S. Campana, M. Campanelli, A. Camplani, A. Campoverde, V. Canale, M. Cano Bret, J. Cantero, T. Cao, M. D. M. Capeans Garrido, I. Caprini, M. Caprini, M. Capua, R. M. Carbone, R. Cardarelli, F. Cardillo, I. Carli, T. Carli, G. Carlino, B. T. Carlson, L. Carminati, R. M. D. Carney, S. Caron, E. Carquin, S. Carrá, G. D. Carrillo-Montoya, J. Carvalho, D. Casadei, M. P. Casado, M. Casolino, D. W. Casper, R. Castelijn, V. Castillo Gimenez, N. F. Castro, A. Catinaccio, J. R. Catmore, A. Cattai, J. Caudron, V. Cavaliere, E. Cavallaro, D. Cavalli, M. Cavalli-Sforza, V. Cavasinni, E. Celebi, F. Ceradini, L. Cerda Alberich, A. S. Cerqueira, A. Cerri, L. Cerrito, F. Cerutti, A. Cervelli, S. A. Cetin, A. Chafaq, D. Chakraborty, S. K. Chan, W. S. Chan, Y. L. Chan, P. Chang, J. D. Chapman, D. G. Charlton, C. C. Chau, C. A. Chavez Barajas, S. Che, S. Cheatham, A. Chegwidden, S. Chekanov, S. V. Chekulaev, G. A. Chelkov, M. A. Chelstowska, C. Chen, H. Chen, S. Chen, S. Chen, X. Chen, Y. Chen, H. C. Cheng, H. J. Cheng, A. Cheplakov, E. Cheremushkina, R. Cherkaoui El Moursli, V. Chernyatin, E. Cheu, L. Chevalier, V. Chiarella, G. Chiarelli, G. Chiodini, A. S. Chisholm, A. Chitan, Y. H. Chiu, M. V. Chizhov, K. Choi, A. R. Chomont, S. Chouridou, V. Christodoulou, D. Chromek-Burckhart, M. C. Chu, J. Chudoba, A. J. Chuinard, J. J. Chwastowski, L. Chytka, A. K. Ciftci, D. Cinca, V. Cindro, I. A. Cioara, C. Ciocca, A. Ciocio, F. Cirotto, Z. H. Citron, M. Citterio, M. Ciubancan, A. Clark, B. L. Clark, M. R. Clark, P. J. Clark, R. N. Clarke, C. Clement, Y. Coadou, M. Cobal, A. Coccaro, J. Cochran, L. Colasurdo, B. Cole, A. P. Colijn, J. Collot, T. Colombo, P. Conde Muiño, E. Coniavitis, S. H. Connell, I. A. Connelly, S. Constantinescu, G. Conti, F. Conventi, M. Cooke, A. M. Cooper-Sarkar, F. Cormier, K. J. R. Cormier, M. Corradi, F. Corriveau, A. Cortes-Gonzalez, G. Cortiana, G. Costa, M. J. Costa, D. Costanzo, G. Cottin, G. Cowan, B. E. Cox, K. Cranmer, S. J. Crawley, R. A. Creager, G. Cree, S. Crépé-Renaudin, F. Crescioli, W. A. Cribbs, M. Cristinziani, V. Croft, G. Crosetti, A. Cueto, T. Cuhadar Donszelmann, A. R. Cukierman, J. Cummings, M. Curatolo, J. Cúth, H. Czirr, P. Czodrowski, G. D’amen, S. D’Auria, L. D’eramo, M. D’Onofrio, M. J. Da Cunha Sargedas De Sousa, C. Da Via, W. Dabrowski, T. Dado, T. Dai, O. Dale, F. Dallaire, C. Dallapiccola, M. Dam, J. R. Dandoy, M. F. Daneri, N. P. Dang, A. C. Daniells, N. S. Dann, M. Danninger, M. Dano Hoffmann, V. Dao, G. Darbo, S. Darmora, J. Dassoulas, A. Dattagupta, T. Daubney, W. Davey, C. David, T. Davidek, M. Davies, D. R. Davis, P. Davison, E. Dawe, I. Dawson, K. De, R. de Asmundis, A. De Benedetti, S. De Castro, S. De Cecco, N. De Groot, P. de Jong, H. De la Torre, F. De Lorenzi, A. De Maria, D. De Pedis, A. De Salvo, U. De Sanctis, A. De Santo, K. De Vasconcelos Corga, J. B. De Vivie De Regie, W. J. Dearnaley, R. Debbe, C. Debenedetti, D. V. Dedovich, N. Dehghanian, I. Deigaard, M. Del Gaudio, J. Del Peso, T. Del Prete, D. Delgove, F. Deliot, C. M. Delitzsch, A. Dell’Acqua, L. Dell’Asta, M. Dell’Orso, M. Della Pietra, D. della Volpe, M. Delmastro, C. Delporte, P. A. Delsart, D. A. DeMarco, S. Demers, M. Demichev, A. Demilly, S. P. Denisov, D. Denysiuk, D. Derendarz, J. E. Derkaoui, F. Derue, P. Dervan, K. Desch, C. Deterre, K. Dette, M. R. Devesa, P. O. Deviveiros, A. Dewhurst, S. Dhaliwal, F. A. Di Bello, A. Di Ciaccio, L. Di Ciaccio, W. K. Di Clemente, C. Di Donato, A. Di Girolamo, B. Di Girolamo, B. Di Micco, R. Di Nardo, K. F. Di Petrillo, A. Di Simone, R. Di Sipio, D. Di Valentino, C. Diaconu, M. Diamond, F. A. Dias, M. A. Diaz, E. B. Diehl, J. Dietrich, S. Díez Cornell, A. Dimitrievska, J. Dingfelder, P. Dita, S. Dita, F. Dittus, F. Djama, T. Djobava, J. I. Djuvsland, M. A. B. do Vale, D. Dobos, M. Dobre, C. Doglioni, J. Dolejsi, Z. Dolezal, M. Donadelli, S. Donati, P. Dondero, J. Donini, J. Dopke, A. Doria, M. T. Dova, A. T. Doyle, E. Drechsler, M. Dris, Y. Du, J. Duarte-Campderros, A. Dubreuil, E. Duchovni, G. Duckeck, A. Ducourthial, O. A. Ducu, D. Duda, A. Dudarev, A. Chr. Dudder, E. M. Duffield, L. Duflot, M. Dührssen, M. Dumancic, A. E. Dumitriu, A. K. Duncan, M. Dunford, H. Duran Yildiz, M. Düren, A. Durglishvili, D. Duschinger, B. Dutta, M. Dyndal, C. Eckardt, K. M. Ecker, R. C. Edgar, T. Eifert, G. Eigen, K. Einsweiler, T. Ekelof, M. El Kacimi, R. El Kosseifi, V. Ellajosyula, M. Ellert, S. Elles, F. Ellinghaus, A. A. Elliot, N. Ellis, J. Elmsheuser, M. Elsing, D. Emeliyanov, Y. Enari, O. C. Endner, J. S. Ennis, J. Erdmann, A. Ereditato, G. Ernis, M. Ernst, S. Errede, M. Escalier, C. Escobar, B. Esposito, O. Estrada Pastor, A. I. Etienvre, E. Etzion, H. Evans, A. Ezhilov, M. Ezzi, F. Fabbri, L. Fabbri, G. Facini, R. M. Fakhrutdinov, S. Falciano, R. J. Falla, J. Faltova, Y. Fang, M. Fanti, A. Farbin, A. Farilla, C. Farina, E. M. Farina, T. Farooque, S. Farrell, S. M. Farrington, P. Farthouat, F. Fassi, P. Fassnacht, D. Fassouliotis, M. Faucci Giannelli, A. Favareto, W. J. Fawcett, L. Fayard, O. L. Fedin, W. Fedorko, S. Feigl, L. Feligioni, C. Feng, E. J. Feng, H. Feng, M. J. Fenton, A. B. Fenyuk, L. Feremenga, P. Fernandez Martinez, S. Fernandez Perez, J. Ferrando, A. Ferrari, P. Ferrari, R. Ferrari, D. E. Ferreira de Lima, A. Ferrer, D. Ferrere, C. Ferretti, F. Fiedler, A. Filipčič, M. Filipuzzi, F. Filthaut, M. Fincke-Keeler, K. D. Finelli, M. C. N. Fiolhais, L. Fiorini, A. Fischer, C. Fischer, J. Fischer, W. C. Fisher, N. Flaschel, I. Fleck, P. Fleischmann, R. R. M. Fletcher, T. Flick, B. M. Flierl, L. R. Flores Castillo, M. J. Flowerdew, G. T. Forcolin, A. Formica, F. A. Förster, A. Forti, A. G. Foster, D. Fournier, H. Fox, S. Fracchia, P. Francavilla, M. Franchini, S. Franchino, D. Francis, L. Franconi, M. Franklin, M. Frate, M. Fraternali, D. Freeborn, S. M. Fressard-Batraneanu, B. Freund, D. Froidevaux, J. A. Frost, C. Fukunaga, T. Fusayasu, J. Fuster, C. Gabaldon, O. Gabizon, A. Gabrielli, A. Gabrielli, G. P. Gach, S. Gadatsch, S. Gadomski, G. Gagliardi, L. G. Gagnon, C. Galea, B. Galhardo, E. J. Gallas, B. J. Gallop, P. Gallus, G. Galster, K. K. Gan, S. Ganguly, Y. Gao, Y. S. Gao, F. M. Garay Walls, C. García, J. E. García Navarro, M. Garcia-Sciveres, R. W. Gardner, N. Garelli, V. Garonne, A. Gascon Bravo, K. Gasnikova, C. Gatti, A. Gaudiello, G. Gaudio, I. L. Gavrilenko, C. Gay, G. Gaycken, E. N. Gazis, C. N. P. Gee, J. Geisen, M. Geisen, M. P. Geisler, K. Gellerstedt, C. Gemme, M. H. Genest, C. Geng, S. Gentile, C. Gentsos, S. George, D. Gerbaudo, A. Gershon, G. Geßner, S. Ghasemi, M. Ghneimat, B. Giacobbe, S. Giagu, P. Giannetti, S. M. Gibson, M. Gignac, M. Gilchriese, D. Gillberg, G. Gilles, D. M. Gingrich, N. Giokaris, M. P. Giordani, F. M. Giorgi, P. F. Giraud, P. Giromini, D. Giugni, F. Giuli, C. Giuliani, M. Giulini, B. K. Gjelsten, S. Gkaitatzis, I. Gkialas, E. L. Gkougkousis, P. Gkountoumis, L. K. Gladilin, C. Glasman, J. Glatzer, P. C. F. Glaysher, A. Glazov, M. Goblirsch-Kolb, J. Godlewski, S. Goldfarb, T. Golling, D. Golubkov, A. Gomes, R. Gonçalo, R. Goncalves Gama, J. Goncalves Pinto Firmino Da Costa, G. Gonella, L. Gonella, A. Gongadze, S. González de la Hoz, S. Gonzalez-Sevilla, L. Goossens, P. A. Gorbounov, H. A. Gordon, I. Gorelov, B. Gorini, E. Gorini, A. Gorišek, A. T. Goshaw, C. Gössling, M. I. Gostkin, C. A. Gottardo, C. R. Goudet, D. Goujdami, A. G. Goussiou, N. Govender, E. Gozani, L. Graber, I. Grabowska-Bold, P. O. J. Gradin, J. Gramling, E. Gramstad, S. Grancagnolo, V. Gratchev, P. M. Gravila, C. Gray, H. M. Gray, Z. D. Greenwood, C. Grefe, K. Gregersen, I. M. Gregor, P. Grenier, K. Grevtsov, J. Griffiths, A. A. Grillo, K. Grimm, S. Grinstein, Ph. Gris, J.-F. Grivaz, S. Groh, E. Gross, J. Grosse-Knetter, G. C. Grossi, Z. J. Grout, A. Grummer, L. Guan, W. Guan, J. Guenther, F. Guescini, D. Guest, O. Gueta, B. Gui, E. Guido, T. Guillemin, S. Guindon, U. Gul, C. Gumpert, J. Guo, W. Guo, Y. Guo, R. Gupta, S. Gupta, G. Gustavino, P. Gutierrez, N. G. Gutierrez Ortiz, C. Gutschow, C. Guyot, M. P. Guzik, C. Gwenlan, C. B. Gwilliam, A. Haas, C. Haber, H. K. Hadavand, N. Haddad, A. Hadef, S. Hageböck, M. Hagihara, H. Hakobyan, M. Haleem, J. Haley, G. Halladjian, G. D. Hallewell, K. Hamacher, P. Hamal, K. Hamano, A. Hamilton, G. N. Hamity, P. G. Hamnett, L. Han, S. Han, K. Hanagaki, K. Hanawa, M. Hance, B. Haney, P. Hanke, J. B. Hansen, J. D. Hansen, M. C. Hansen, P. H. Hansen, K. Hara, A. S. Hard, T. Harenberg, F. Hariri, S. Harkusha, R. D. Harrington, P. F. Harrison, N. M. Hartmann, M. Hasegawa, Y. Hasegawa, A. Hasib, S. Hassani, S. Haug, R. Hauser, L. Hauswald, L. B. Havener, M. Havranek, C. M. Hawkes, R. J. Hawkings, D. Hayakawa, D. Hayden, C. P. Hays, J. M. Hays, H. S. Hayward, S. J. Haywood, S. J. Head, T. Heck, V. Hedberg, L. Heelan, K. K. Heidegger, S. Heim, T. Heim, B. Heinemann, J. J. Heinrich, L. Heinrich, C. Heinz, J. Hejbal, L. Helary, A. Held, S. Hellman, C. Helsens, R. C. W. Henderson, Y. Heng, S. Henkelmann, A. M. Henriques Correia, S. Henrot-Versille, G. H. Herbert, H. Herde, V. Herget, Y. Hernández Jiménez, H. Herr, G. Herten, R. Hertenberger, L. Hervas, T. C. Herwig, G. G. Hesketh, N. P. Hessey, J. W. Hetherly, S. Higashino, E. Higón-Rodriguez, E. Hill, J. C. Hill, K. H. Hiller, S. J. Hillier, M. Hils, I. Hinchliffe, M. Hirose, D. Hirschbuehl, B. Hiti, O. Hladik, X. Hoad, J. Hobbs, N. Hod, M. C. Hodgkinson, P. Hodgson, A. Hoecker, M. R. Hoeferkamp, F. Hoenig, D. Hohn, T. R. Holmes, M. Homann, S. Honda, T. Honda, T. M. Hong, B. H. Hooberman, W. H. Hopkins, Y. Horii, A. J. Horton, J.-Y. Hostachy, S. Hou, A. Hoummada, J. Howarth, J. Hoya, M. Hrabovsky, J. Hrdinka, I. Hristova, J. Hrivnac, T. Hryn’ova, A. Hrynevich, P. J. Hsu, S.-C. Hsu, Q. Hu, S. Hu, Y. Huang, Z. Hubacek, F. Hubaut, F. Huegging, T. B. Huffman, E. W. Hughes, G. Hughes, M. Huhtinen, P. Huo, N. Huseynov, J. Huston, J. Huth, G. Iacobucci, G. Iakovidis, I. Ibragimov, L. Iconomidou-Fayard, Z. Idrissi, P. Iengo, O. Igonkina, T. Iizawa, Y. Ikegami, M. Ikeno, Y. Ilchenko, D. Iliadis, N. Ilic, G. Introzzi, P. Ioannou, M. Iodice, K. Iordanidou, V. Ippolito, M. F. Isacson, N. Ishijima, M. Ishino, M. Ishitsuka, C. Issever, S. Istin, F. Ito, J. M. Iturbe Ponce, R. Iuppa, H. Iwasaki, J. M. Izen, V. Izzo, S. Jabbar, P. Jackson, R. M. Jacobs, V. Jain, K. B. Jakobi, K. Jakobs, S. Jakobsen, T. Jakoubek, D. O. Jamin, D. K. Jana, R. Jansky, J. Janssen, M. Janus, P. A. Janus, G. Jarlskog, N. Javadov, T. Javůrek, M. Javurkova, F. Jeanneau, L. Jeanty, J. Jejelava, A. Jelinskas, P. Jenni, C. Jeske, S. Jézéquel, H. Ji, J. Jia, H. Jiang, Y. Jiang, Z. Jiang, S. Jiggins, J. Jimenez Pena, S. Jin, A. Jinaru, O. Jinnouchi, H. Jivan, P. Johansson, K. A. Johns, C. A. Johnson, W. J. Johnson, K. Jon-And, R. W. L. Jones, S. D. Jones, S. Jones, T. J. Jones, J. Jongmanns, P. M. Jorge, J. Jovicevic, X. Ju, A. Juste Rozas, M. K. Köhler, A. Kaczmarska, M. Kado, H. Kagan, M. Kagan, S. J. Kahn, T. Kaji, E. Kajomovitz, C. W. Kalderon, A. Kaluza, S. Kama, A. Kamenshchikov, N. Kanaya, L. Kanjir, V. A. Kantserov, J. Kanzaki, B. Kaplan, L. S. Kaplan, D. Kar, K. Karakostas, N. Karastathis, M. J. Kareem, E. Karentzos, S. N. Karpov, Z. M. Karpova, K. Karthik, V. Kartvelishvili, A. N. Karyukhin, K. Kasahara, L. Kashif, R. D. Kass, A. Kastanas, Y. Kataoka, C. Kato, A. Katre, J. Katzy, K. Kawade, K. Kawagoe, T. Kawamoto, G. Kawamura, E. F. Kay, V. F. Kazanin, R. Keeler, R. Kehoe, J. S. Keller, J. J. Kempster, J Kendrick, H. Keoshkerian, O. Kepka, B. P. Kerševan, S. Kersten, R. A. Keyes, M. Khader, F. Khalil-zada, A. Khanov, A. G. Kharlamov, T. Kharlamova, A. Khodinov, T. J. Khoo, V. Khovanskiy, E. Khramov, J. Khubua, S. Kido, C. R. Kilby, H. Y. Kim, S. H. Kim, Y. K. Kim, N. Kimura, O. M. Kind, B. T. King, D. Kirchmeier, J. Kirk, A. E. Kiryunin, T. Kishimoto, D. Kisielewska, V. Kitali, K. Kiuchi, O. Kivernyk, E. Kladiva, T. Klapdor-Kleingrothaus, M. H. Klein, M. Klein, U. Klein, K. Kleinknecht, P. Klimek, A. Klimentov, R. Klingenberg, T. Klingl, T. Klioutchnikova, E.-E. Kluge, P. Kluit, S. Kluth, E. Kneringer, E. B. F. G. Knoops, A. Knue, A. Kobayashi, D. Kobayashi, T. Kobayashi, M. Kobel, M. Kocian, P. Kodys, T. Koffas, E. Koffeman, N. M. Köhler, T. Koi, M. Kolb, I. Koletsou, A. A. Komar, Y. Komori, T. Kondo, N. Kondrashova, K. Köneke, A. C. König, T. Kono, R. Konoplich, N. Konstantinidis, R. Kopeliansky, S. Koperny, A. K. Kopp, K. Korcyl, K. Kordas, A. Korn, A. A. Korol, I. Korolkov, E. V. Korolkova, O. Kortner, S. Kortner, T. Kosek, V. V. Kostyukhin, A. Kotwal, A. Koulouris, A. Kourkoumeli-Charalampidi, C. Kourkoumelis, E. Kourlitis, V. Kouskoura, A. B. Kowalewska, R. Kowalewski, T. Z. Kowalski, C. Kozakai, W. Kozanecki, A. S. Kozhin, V. A. Kramarenko, G. Kramberger, D. Krasnopevtsev, M. W. Krasny, A. Krasznahorkay, D. Krauss, J. A. Kremer, J. Kretzschmar, K. Kreutzfeldt, P. Krieger, K. Krizka, K. Kroeninger, H. Kroha, J. Kroll, J. Kroll, J. Kroseberg, J. Krstic, U. Kruchonak, H. Krüger, N. Krumnack, M. C. Kruse, T. Kubota, H. Kucuk, S. Kuday, J. T. Kuechler, S. Kuehn, A. Kugel, F. Kuger, T. Kuhl, V. Kukhtin, R. Kukla, Y. Kulchitsky, S. Kuleshov, Y. P. Kulinich, M. Kuna, T. Kunigo, A. Kupco, T. Kupfer, O. Kuprash, H. Kurashige, L. L. Kurchaninov, Y. A. Kurochkin, M. G. Kurth, V. Kus, E. S. Kuwertz, M. Kuze, J. Kvita, T. Kwan, D. Kyriazopoulos, A. La Rosa, J. L. La Rosa Navarro, L. La Rotonda, C. Lacasta, F. Lacava, J. Lacey, H. Lacker, D. Lacour, E. Ladygin, R. Lafaye, B. Laforge, T. Lagouri, S. Lai, S. Lammers, W. Lampl, E. Lançon, U. Landgraf, M. P. J. Landon, M. C. Lanfermann, V. S. Lang, J. C. Lange, R. J. Langenberg, A. J. Lankford, F. Lanni, K. Lantzsch, A. Lanza, A. Lapertosa, S. Laplace, J. F. Laporte, T. Lari, F. Lasagni Manghi, M. Lassnig, P. Laurelli, W. Lavrijsen, A. T. Law, P. Laycock, T. Lazovich, M. Lazzaroni, B. Le, O. Le Dortz, E. Le Guirriec, E. P. Le Quilleuc, M. LeBlanc, T. LeCompte, F. Ledroit-Guillon, C. A. Lee, G. R. Lee, S. C. Lee, L. Lee, B. Lefebvre, G. Lefebvre, M. Lefebvre, F. Legger, C. Leggett, A. Lehan, G. Lehmann Miotto, X. Lei, W. A. Leight, M. A. L. Leite, R. Leitner, D. Lellouch, B. Lemmer, K. J. C. Leney, T. Lenz, B. Lenzi, R. Leone, S. Leone, C. Leonidopoulos, G. Lerner, C. Leroy, A. A. J. Lesage, C. G. Lester, M. Levchenko, J. Levêque, D. Levin, L. J. Levinson, M. Levy, D. Lewis, B. Li, Changqiao Li, H. Li, L. Li, Q. Li, S. Li, X. Li, Y. Li, Z. Liang, B. Liberti, A. Liblong, K. Lie, J. Liebal, W. Liebig, A. Limosani, S. C. Lin, T. H. Lin, B. E. Lindquist, A. E. Lionti, E. Lipeles, A. Lipniacka, M. Lisovyi, T. M. Liss, A. Lister, A. M. Litke, B. Liu, H. Liu, H. Liu, J. K. K. Liu, J. Liu, J. B. Liu, K. Liu, L. Liu, M. Liu, Y. L. Liu, Y. Liu, M. Livan, A. Lleres, J. Llorente Merino, S. L. Lloyd, C. Y. Lo, F. Lo Sterzo, E. M. Lobodzinska, P. Loch, F. K. Loebinger, A. Loesle, K. M. Loew, A. Loginov, T. Lohse, K. Lohwasser, M. Lokajicek, B. A. Long, J. D. Long, R. E. Long, L. Longo, K. A. Looper, J. A. Lopez, D. Lopez Mateos, I. Lopez Paz, A. Lopez Solis, J. Lorenz, N. Lorenzo Martinez, M. Losada, P. J. Lösel, X. Lou, A. Lounis, J. Love, P. A. Love, H. Lu, N. Lu, Y. J. Lu, H. J. Lubatti, C. Luci, A. Lucotte, C. Luedtke, F. Luehring, W. Lukas, L. Luminari, O. Lundberg, B. Lund-Jensen, P. M. Luzi, D. Lynn, R. Lysak, E. Lytken, V. Lyubushkin, H. Ma, L. L. Ma, Y. Ma, G. Maccarrone, A. Macchiolo, C. M. Macdonald, B. Maček, J. Machado Miguens, D. Madaffari, R. Madar, W. F. Mader, A. Madsen, J. Maeda, S. Maeland, T. Maeno, A. S. Maevskiy, E. Magradze, J. Mahlstedt, C. Maiani, C. Maidantchik, A. A. Maier, T. Maier, A. Maio, O. Majersky, S. Majewski, Y. Makida, N. Makovec, B. Malaescu, Pa. Malecki, V. P. Maleev, F. Malek, U. Mallik, D. Malon, C. Malone, S. Maltezos, S. Malyukov, J. Mamuzic, G. Mancini, L. Mandelli, I. Mandić, J. Maneira, L. Manhaes de Andrade Filho, J. Manjarres Ramos, A. Mann, A. Manousos, B. Mansoulie, J. D. Mansour, R. Mantifel, M. Mantoani, S. Manzoni, L. Mapelli, G. Marceca, L. March, L. Marchese, G. Marchiori, M. Marcisovsky, M. Marjanovic, D. E. Marley, F. Marroquim, S. P. Marsden, Z. Marshall, M. U. F Martensson, S. Marti-Garcia, C. B. Martin, T. A. Martin, V. J. Martin, B. Martin dit Latour, M. Martinez, V. I. Martinez Outschoorn, S. Martin-Haugh, V. S. Martoiu, A. C. Martyniuk, A. Marzin, L. Masetti, T. Mashimo, R. Mashinistov, J. Masik, A. L. Maslennikov, L. Massa, P. Mastrandrea, A. Mastroberardino, T. Masubuchi, P. Mättig, J. Maurer, S. J. Maxfield, D. A. Maximov, R. Mazini, I. Maznas, S. M. Mazza, N. C. Mc Fadden, G. Mc Goldrick, S. P. Mc Kee, A. McCarn, R. L. McCarthy, T. G. McCarthy, L. I. McClymont, E. F. McDonald, J. A. Mcfayden, G. Mchedlidze, S. J. McMahon, P. C. McNamara, R. A. McPherson, S. Meehan, T. J. Megy, S. Mehlhase, A. Mehta, T. Meideck, K. Meier, B. Meirose, D. Melini, B. R. Mellado Garcia, J. D. Mellenthin, M. Melo, F. Meloni, S. B. Menary, L. Meng, X. T. Meng, A. Mengarelli, S. Menke, E. Meoni, S. Mergelmeyer, P. Mermod, L. Merola, C. Meroni, F. S. Merritt, A. Messina, J. Metcalfe, A. S. Mete, C. Meyer, J.-P. Meyer, J. Meyer, H. Meyer Zu Theenhausen, F. Miano, R. P. Middleton, S. Miglioranzi, L. Mijović, G. Mikenberg, M. Mikestikova, M. Mikuž, M. Milesi, A. Milic, D. W. Miller, C. Mills, A. Milov, D. A. Milstead, A. A. Minaenko, Y. Minami, I. A. Minashvili, A. I. Mincer, B. Mindur, M. Mineev, Y. Minegishi, Y. Ming, L. M. Mir, K. P. Mistry, T. Mitani, J. Mitrevski, V. A. Mitsou, A. Miucci, P. S. Miyagawa, A. Mizukami, J. U. Mjörnmark, T. Mkrtchyan, M. Mlynarikova, T. Moa, K. Mochizuki, P. Mogg, S. Mohapatra, S. Molander, R. Moles-Valls, R. Monden, M. C. Mondragon, K. Mönig, J. Monk, E. Monnier, A. Montalbano, J. Montejo Berlingen, F. Monticelli, S. Monzani, R. W. Moore, N. Morange, D. Moreno, M. Moreno Llácer, P. Morettini, S. Morgenstern, D. Mori, T. Mori, M. Morii, M. Morinaga, V. Morisbak, A. K. Morley, G. Mornacchi, J. D. Morris, L. Morvaj, P. Moschovakos, M. Mosidze, H. J. Moss, J. Moss, K. Motohashi, R. Mount, E. Mountricha, E. J. W. Moyse, S. Muanza, R. D. Mudd, F. Mueller, J. Mueller, R. S. P. Mueller, D. Muenstermann, P. Mullen, G. A. Mullier, F. J. Munoz Sanchez, W. J. Murray, H. Musheghyan, M. Muškinja, A. G. Myagkov, M. Myska, B. P. Nachman, O. Nackenhorst, K. Nagai, R. Nagai, K. Nagano, Y. Nagasaka, K. Nagata, M. Nagel, E. Nagy, A. M. Nairz, Y. Nakahama, K. Nakamura, T. Nakamura, I. Nakano, R. F. Naranjo Garcia, R. Narayan, D. I. Narrias Villar, I. Naryshkin, T. Naumann, G. Navarro, R. Nayyar, H. A. Neal, P. Yu. Nechaeva, T. J. Neep, A. Negri, M. Negrini, S. Nektarijevic, C. Nellist, A. Nelson, M. E. Nelson, S. Nemecek, P. Nemethy, M. Nessi, M. S. Neubauer, M. Neumann, P. R. Newman, T. Y. Ng, T. Nguyen Manh, R. B. Nickerson, R. Nicolaidou, J. Nielsen, V. Nikolaenko, I. Nikolic-Audit, K. Nikolopoulos, J. K. Nilsen, P. Nilsson, Y. Ninomiya, A. Nisati, N. Nishu, R. Nisius, I. Nitsche, T. Nobe, Y. Noguchi, M. Nomachi, I. Nomidis, M. A. Nomura, T. Nooney, M. Nordberg, N. Norjoharuddeen, O. Novgorodova, S. Nowak, M. Nozaki, L. Nozka, K. Ntekas, E. Nurse, F. Nuti, K. O’connor, D. C. O’Neil, A. A. O’Rourke, V. O’Shea, F. G. Oakham, H. Oberlack, T. Obermann, J. Ocariz, A. Ochi, I. Ochoa, J. P. Ochoa-Ricoux, S. Oda, S. Odaka, H. Ogren, A. Oh, S. H. Oh, C. C. Ohm, H. Ohman, H. Oide, H. Okawa, Y. Okumura, T. Okuyama, A. Olariu, L. F. Oleiro Seabra, S. A. Olivares Pino, D. Oliveira Damazio, A. Olszewski, J. Olszowska, A. Onofre, K. Onogi, P. U. E. Onyisi, M. J. Oreglia, Y. Oren, D. Orestano, N. Orlando, R. S. Orr, B. Osculati, R. Ospanov, G. Otero y Garzon, H. Otono, M. Ouchrif, F. Ould-Saada, A. Ouraou, K. P. Oussoren, Q. Ouyang, M. Owen, R. E. Owen, V. E. Ozcan, N. Ozturk, K. Pachal, A. Pacheco Pages, L. Pacheco Rodriguez, C. Padilla Aranda, S. Pagan Griso, M. Paganini, F. Paige, G. Palacino, S. Palazzo, S. Palestini, M. Palka, D. Pallin, E. St. Panagiotopoulou, I. Panagoulias, C. E. Pandini, J. G. Panduro Vazquez, P. Pani, S. Panitkin, D. Pantea, L. Paolozzi, Th. D. Papadopoulou, K. Papageorgiou, A. Paramonov, D. Paredes Hernandez, A. J. Parker, M. A. Parker, K. A. Parker, F. Parodi, J. A. Parsons, U. Parzefall, V. R. Pascuzzi, J. M. Pasner, E. Pasqualucci, S. Passaggio, Fr. Pastore, S. Pataraia, J. R. Pater, T. Pauly, B. Pearson, S. Pedraza Lopez, R. Pedro, S. V. Peleganchuk, O. Penc, C. Peng, H. Peng, J. Penwell, B. S. Peralva, M. M. Perego, D. V. Perepelitsa, L. Perini, H. Pernegger, S. Perrella, R. Peschke, V. D. Peshekhonov, K. Peters, R. F. Y. Peters, B. A. Petersen, T. C. Petersen, E. Petit, A. Petridis, C. Petridou, P. Petroff, E. Petrolo, M. Petrov, F. Petrucci, N. E. Pettersson, A. Peyaud, R. Pezoa, F. H. Phillips, P. W. Phillips, G. Piacquadio, E. Pianori, A. Picazio, E. Piccaro, M. A. Pickering, R. Piegaia, J. E. Pilcher, A. D. Pilkington, A. W. J. Pin, M. Pinamonti, J. L. Pinfold, H. Pirumov, M. Pitt, L. Plazak, M.-A. Pleier, V. Pleskot, E. Plotnikova, D. Pluth, P. Podberezko, R. Poettgen, R. Poggi, L. Poggioli, D. Pohl, G. Polesello, A. Poley, A. Policicchio, R. Polifka, A. Polini, C. S. Pollard, V. Polychronakos, K. Pommès, D. Ponomarenko, L. Pontecorvo, B. G. Pope, G. A. Popeneciu, A. Poppleton, S. Pospisil, K. Potamianos, I. N. Potrap, C. J. Potter, G. Poulard, T. Poulsen, J. Poveda, M. E. Pozo Astigarraga, P. Pralavorio, A. Pranko, S. Prell, D. Price, L. E. Price, M. Primavera, S. Prince, N. Proklova, K. Prokofiev, F. Prokoshin, S. Protopopescu, J. Proudfoot, M. Przybycien, A. Puri, P. Puzo, J. Qian, G. Qin, Y. Qin, A. Quadt, M. Queitsch-Maitland, D. Quilty, S. Raddum, V. Radeka, V. Radescu, S. K. Radhakrishnan, P. Radloff, P. Rados, F. Ragusa, G. Rahal, J. A. Raine, S. Rajagopalan, C. Rangel-Smith, T. Rashid, S. Raspopov, M. G. Ratti, D. M. Rauch, F. Rauscher, S. Rave, I. Ravinovich, J. H. Rawling, M. Raymond, A. L. Read, N. P. Readioff, M. Reale, D. M. Rebuzzi, A. Redelbach, G. Redlinger, R. Reece, R. G. Reed, K. Reeves, L. Rehnisch, J. Reichert, A. Reiss, C. Rembser, H. Ren, M. Rescigno, S. Resconi, E. D. Resseguie, S. Rettie, E. Reynolds, O. L. Rezanova, P. Reznicek, R. Rezvani, R. Richter, S. Richter, E. Richter-Was, O. Ricken, M. Ridel, P. Rieck, C. J. Riegel, J. Rieger, O. Rifki, M. Rijssenbeek, A. Rimoldi, M. Rimoldi, L. Rinaldi, G. Ripellino, B. Ristić, E. Ritsch, I. Riu, F. Rizatdinova, E. Rizvi, C. Rizzi, R. T. Roberts, S. H. Robertson, A. Robichaud-Veronneau, D. Robinson, J. E. M. Robinson, A. Robson, E. Rocco, C. Roda, Y. Rodina, S. Rodriguez Bosca, A. Rodriguez Perez, D. Rodriguez Rodriguez, S. Roe, C. S. Rogan, O. Røhne, J. Roloff, A. Romaniouk, M. Romano, S. M. Romano Saez, E. Romero Adam, N. Rompotis, M. Ronzani, L. Roos, S. Rosati, K. Rosbach, P. Rose, N.-A. Rosien, E. Rossi, L. P. Rossi, J. H. N. Rosten, R. Rosten, M. Rotaru, I. Roth, J. Rothberg, D. Rousseau, A. Rozanov, Y. Rozen, X. Ruan, F. Rubbo, F. Rühr, A. Ruiz-Martinez, Z. Rurikova, N. A. Rusakovich, H. L. Russell, J. P. Rutherfoord, N. Ruthmann, Y. F. Ryabov, M. Rybar, G. Rybkin, S. Ryu, A. Ryzhov, G. F. Rzehorz, A. F. Saavedra, G. Sabato, S. Sacerdoti, H.F-W. Sadrozinski, R. Sadykov, F. Safai Tehrani, P. Saha, M. Sahinsoy, M. Saimpert, M. Saito, T. Saito, H. Sakamoto, Y. Sakurai, G. Salamanna, J. E. Salazar Loyola, D. Salek, P. H. Sales De Bruin, D. Salihagic, A. Salnikov, J. Salt, D. Salvatore, F. Salvatore, A. Salvucci, A. Salzburger, D. Sammel, D. Sampsonidis, D. Sampsonidou, J. Sánchez, V. Sanchez Martinez, A. Sanchez Pineda, H. Sandaker, R. L. Sandbach, C. O. Sander, M. Sandhoff, C. Sandoval, D. P. C. Sankey, M. Sannino, A. Sansoni, C. Santoni, R. Santonico, H. Santos, I. Santoyo Castillo, A. Sapronov, J. G. Saraiva, B. Sarrazin, O. Sasaki, K. Sato, E. Sauvan, G. Savage, P. Savard, N. Savic, C. Sawyer, L. Sawyer, J. Saxon, C. Sbarra, A. Sbrizzi, T. Scanlon, D. A. Scannicchio, M. Scarcella, V. Scarfone, J. Schaarschmidt, P. Schacht, B. M. Schachtner, D. Schaefer, L. Schaefer, R. Schaefer, J. Schaeffer, S. Schaepe, S. Schaetzel, U. Schäfer, A. C. Schaffer, D. Schaile, R. D. Schamberger, V. Scharf, V. A. Schegelsky, D. Scheirich, M. Schernau, C. Schiavi, S. Schier, L. K. Schildgen, C. Schillo, M. Schioppa, S. Schlenker, K. R. Schmidt-Sommerfeld, K. Schmieden, C. Schmitt, S. Schmitt, S. Schmitz, U. Schnoor, L. Schoeffel, A. Schoening, B. D. Schoenrock, E. Schopf, M. Schott, J. F. P. Schouwenberg, J. Schovancova, S. Schramm, N. Schuh, A. Schulte, M. J. Schultens, H.-C. Schultz-Coulon, H. Schulz, M. Schumacher, B. A. Schumm, Ph. Schune, A. Schwartzman, T. A. Schwarz, H. Schweiger, Ph. Schwemling, R. Schwienhorst, J. Schwindling, A. Sciandra, G. Sciolla, F. Scuri, F. Scutti, J. Searcy, P. Seema, S. C. Seidel, A. Seiden, J. M. Seixas, G. Sekhniaidze, K. Sekhon, S. J. Sekula, N. Semprini-Cesari, S. Senkin, C. Serfon, L. Serin, L. Serkin, M. Sessa, R. Seuster, H. Severini, T. Sfiligoj, F. Sforza, A. Sfyrla, E. Shabalina, N. W. Shaikh, L. Y. Shan, R. Shang, J. T. Shank, M. Shapiro, P. B. Shatalov, K. Shaw, S. M. Shaw, A. Shcherbakova, C. Y. Shehu, Y. Shen, N. Sherafati, P. Sherwood, L. Shi, S. Shimizu, C. O. Shimmin, M. Shimojima, I. P. J. Shipsey, S. Shirabe, M. Shiyakova, J. Shlomi, A. Shmeleva, D. Shoaleh Saadi, M. J. Shochet, S. Shojaii, D. R. Shope, S. Shrestha, E. Shulga, M. A. Shupe, P. Sicho, A. M. Sickles, P. E. Sidebo, E. Sideras Haddad, O. Sidiropoulou, A. Sidoti, F. Siegert, Dj. Sijacki, J. Silva, S. B. Silverstein, V. Simak, Lj. Simic, S. Simion, E. Simioni, B. Simmons, M. Simon, P. Sinervo, N. B. Sinev, M. Sioli, G. Siragusa, I. Siral, S. Yu. Sivoklokov, J. Sjölin, M. B. Skinner, P. Skubic, M. Slater, T. Slavicek, M. Slawinska, K. Sliwa, R. Slovak, V. Smakhtin, B. H. Smart, J. Smiesko, N. Smirnov, S. Yu. Smirnov, Y. Smirnov, L. N. Smirnova, O. Smirnova, J. W. Smith, M. N. K. Smith, R. W. Smith, M. Smizanska, K. Smolek, A. A. Snesarev, I. M. Snyder, S. Snyder, R. Sobie, F. Socher, A. Soffer, D. A. Soh, G. Sokhrannyi, C. A. Solans Sanchez, M. Solar, E. Yu. Soldatov, U. Soldevila, A. A. Solodkov, A. Soloshenko, O. V. Solovyanov, V. Solovyev, P. Sommer, H. Son, A. Sopczak, D. Sosa, C. L. Sotiropoulou, R. Soualah, A. M. Soukharev, D. South, B. C. Sowden, S. Spagnolo, M. Spalla, M. Spangenberg, F. Spanò, D. Sperlich, F. Spettel, T. M. Spieker, R. Spighi, G. Spigo, L. A. Spiller, M. Spousta, R. D. St. Denis, A. Stabile, R. Stamen, S. Stamm, E. Stanecka, R. W. Stanek, C. Stanescu, M. M. Stanitzki, B. S. Stapf, S. Stapnes, E. A. Starchenko, G. H. Stark, J. Stark, S. H Stark, P. Staroba, P. Starovoitov, S. Stärz, R. Staszewski, P. Steinberg, B. Stelzer, H. J. Stelzer, O. Stelzer-Chilton, H. Stenzel, G. A. Stewart, M. C. Stockton, M. Stoebe, G. Stoicea, P. Stolte, S. Stonjek, A. R. Stradling, A. Straessner, M. E. Stramaglia, J. Strandberg, S. Strandberg, M. Strauss, P. Strizenec, R. Ströhmer, D. M. Strom, R. Stroynowski, A. Strubig, S. A. Stucci, B. Stugu, N. A. Styles, D. Su, J. Su, S. Suchek, Y. Sugaya, M. Suk, V. V. Sulin, DMS Sultan, S. Sultansoy, T. Sumida, S. Sun, X. Sun, K. Suruliz, C. J. E. Suster, M. R. Sutton, S. Suzuki, M. Svatos, M. Swiatlowski, S. P. Swift, I. Sykora, T. Sykora, D. Ta, K. Tackmann, J. Taenzer, A. Taffard, R. Tafirout, N. Taiblum, H. Takai, R. Takashima, E. H. Takasugi, T. Takeshita, Y. Takubo, M. Talby, A. A. Talyshev, J. Tanaka, M. Tanaka, R. Tanaka, S. Tanaka, R. Tanioka, B. B. Tannenwald, S. Tapia Araya, S. Tapprogge, S. Tarem, G. F. Tartarelli, P. Tas, M. Tasevsky, T. Tashiro, E. Tassi, A. Tavares Delgado, Y. Tayalati, A. C. Taylor, G. N. Taylor, P. T. E. Taylor, W. Taylor, P. Teixeira-Dias, D. Temple, H. Ten Kate, P. K. Teng, J. J. Teoh, F. Tepel, S. Terada, K. Terashi, J. Terron, S. Terzo, M. Testa, R. J. Teuscher, T. Theveneaux-Pelzer, J. P. Thomas, J. Thomas-Wilsker, P. D. Thompson, A. S. Thompson, L. A. Thomsen, E. Thomson, M. J. Tibbetts, R. E. Ticse Torres, V. O. Tikhomirov, Yu. A. Tikhonov, S. Timoshenko, P. Tipton, S. Tisserant, K. Todome, S. Todorova-Nova, J. Tojo, S. Tokár, K. Tokushuku, E. Tolley, L. Tomlinson, M. Tomoto, L. Tompkins, K. Toms, B. Tong, P. Tornambe, E. Torrence, H. Torres, E. Torró Pastor, J. Toth, F. Touchard, D. R. Tovey, C. J. Treado, T. Trefzger, F. Tresoldi, A. Tricoli, I. M. Trigger, S. Trincaz-Duvoid, M. F. Tripiana, W. Trischuk, B. Trocmé, A. Trofymov, C. Troncon, M. Trottier-McDonald, M. Trovatelli, L. Truong, M. Trzebinski, A. Trzupek, K. W. Tsang, J.C-L. Tseng, P. V. Tsiareshka, G. Tsipolitis, N. Tsirintanis, S. Tsiskaridze, V. Tsiskaridze, E. G. Tskhadadze, K. M. Tsui, I. I. Tsukerman, V. Tsulaia, S. Tsuno, D. Tsybychev, Y. Tu, A. Tudorache, V. Tudorache, T. T. Tulbure, A. N. Tuna, S. A. Tupputi, S. Turchikhin, D. Turgeman, I. Turk Cakir, R. Turra, P. M. Tuts, G. Ucchielli, I. Ueda, M. Ughetto, F. Ukegawa, G. Unal, A. Undrus, G. Unel, F. C. Ungaro, Y. Unno, C. Unverdorben, J. Urban, P. Urquijo, P. Urrejola, G. Usai, J. Usui, L. Vacavant, V. Vacek, B. Vachon, A. Vaidya, C. Valderanis, E. Valdes Santurio, S. Valentinetti, A. Valero, L. Valéry, S. Valkar, A. Vallier, J. A. Valls Ferrer, W. Van Den Wollenberg, H. van der Graaf, P. van Gemmeren, J. Van Nieuwkoop, I. van Vulpen, M. C. van Woerden, M. Vanadia, W. Vandelli, A. Vaniachine, P. Vankov, G. Vardanyan, R. Vari, E. W. Varnes, C. Varni, T. Varol, D. Varouchas, A. Vartapetian, K. E. Varvell, J. G. Vasquez, G. A. Vasquez, F. Vazeille, T. Vazquez Schroeder, J. Veatch, V. Veeraraghavan, L. M. Veloce, F. Veloso, S. Veneziano, A. Ventura, M. Venturi, N. Venturi, A. Venturini, V. Vercesi, M. Verducci, W. Verkerke, A. T. Vermeulen, J. C. Vermeulen, M. C. Vetterli, N. Viaux Maira, O. Viazlo, I. Vichou, T. Vickey, O. E. Vickey Boeriu, G. H. A. Viehhauser, S. Viel, L. Vigani, M. Villa, M. Villaplana Perez, E. Vilucchi, M. G. Vincter, V. B. Vinogradov, A. Vishwakarma, C. Vittori, I. Vivarelli, S. Vlachos, M. Vlasak, M. Vogel, P. Vokac, G. Volpi, H. von der Schmitt, E. von Toerne, V. Vorobel, K. Vorobev, M. Vos, R. Voss, J. H. Vossebeld, N. Vranjes, M. Vranjes Milosavljevic, V. Vrba, M. Vreeswijk, R. Vuillermet, I. Vukotic, P. Wagner, W. Wagner, J. Wagner-Kuhr, H. Wahlberg, S. Wahrmund, J. Wakabayashi, J. Walder, R. Walker, W. Walkowiak, V. Wallangen, C. Wang, C. Wang, F. Wang, H. Wang, H. Wang, J. Wang, J. Wang, Q. Wang, R. Wang, S. M. Wang, T. Wang, W. Wang, W. Wang, Z. Wang, C. Wanotayaroj, A. Warburton, C. P. Ward, D. R. Wardrope, A. Washbrook, P. M. Watkins, A. T. Watson, M. F. Watson, G. Watts, S. Watts, B. M. Waugh, A. F. Webb, S. Webb, M. S. Weber, S. W. Weber, S. A. Weber, J. S. Webster, A. R. Weidberg, B. Weinert, J. Weingarten, M. Weirich, C. Weiser, H. Weits, P. S. Wells, T. Wenaus, T. Wengler, S. Wenig, N. Wermes, M. D. Werner, P. Werner, M. Wessels, K. Whalen, N. L. Whallon, A. M. Wharton, A. S. White, A. White, M. J. White, R. White, D. Whiteson, B. W. Whitmore, F. J. Wickens, W. Wiedenmann, M. Wielers, C. Wiglesworth, L. A. M. Wiik-Fuchs, A. Wildauer, F. Wilk, H. G. Wilkens, H. H. Williams, S. Williams, C. Willis, S. Willocq, J. A. Wilson, I. Wingerter-Seez, E. Winkels, F. Winklmeier, O. J. Winston, B. T. Winter, M. Wittgen, M. Wobisch, T. M. H. Wolf, R. Wolff, M. W. Wolter, H. Wolters, V. W. S. Wong, S. D. Worm, B. K. Wosiek, J. Wotschack, K. W. Wozniak, M. Wu, S. L. Wu, X. Wu, Y. Wu, T. R. Wyatt, B. M. Wynne, S. Xella, Z. Xi, L. Xia, D. Xu, L. Xu, T. Xu, B. Yabsley, S. Yacoob, D. Yamaguchi, Y. Yamaguchi, A. Yamamoto, S. Yamamoto, T. Yamanaka, M. Yamatani, K. Yamauchi, Y. Yamazaki, Z. Yan, H. Yang, H. Yang, Y. Yang, Z. Yang, W.-M. Yao, Y. C. Yap, Y. Yasu, E. Yatsenko, K. H. Yau Wong, J. Ye, S. Ye, I. Yeletskikh, E. Yigitbasi, E. Yildirim, K. Yorita, K. Yoshihara, C. Young, C. J. S. Young, J. Yu, J. Yu, S. P. Y. Yuen, I. Yusuff, B. Zabinski, G. Zacharis, R. Zaidan, A. M. Zaitsev, N. Zakharchuk, J. Zalieckas, A. Zaman, S. Zambito, D. Zanzi, C. Zeitnitz, G. Zemaityte, A. Zemla, J. C. Zeng, Q. Zeng, O. Zenin, T. Ženiš, D. Zerwas, D. Zhang, F. Zhang, G. Zhang, H. Zhang, J. Zhang, L. Zhang, L. Zhang, M. Zhang, P. Zhang, R. Zhang, R. Zhang, X. Zhang, Y. Zhang, Z. Zhang, X. Zhao, Y. Zhao, Z. Zhao, A. Zhemchugov, B. Zhou, C. Zhou, L. Zhou, M. Zhou, M. Zhou, N. Zhou, C. G. Zhu, H. Zhu, J. Zhu, Y. Zhu, X. Zhuang, K. Zhukov, A. Zibell, D. Zieminska, N. I. Zimine, C. Zimmermann, S. Zimmermann, Z. Zinonos, M. Zinser, M. Ziolkowski, L. Živković, G. Zobernig, A. Zoccoli, R. Zou, M. zur Nedden, L. Zwalinski, ATLAS Collaboration

**Affiliations:** 1 Department of Physics, University of Adelaide, Adelaide, Australia; 2 Physics Department, SUNY Albany, Albany, NY USA; 3 Department of Physics, University of Alberta, Edmonton, AB Canada; 4 Department of Physics, Ankara University, Ankara, Turkey; 5 Istanbul Aydin University, Istanbul, Turkey; 6 Division of Physics, TOBB University of Economics and Technology, Ankara, Turkey; 7 LAPP, CNRS/IN2P3 and Université Savoie Mont Blanc, Annecy-le-Vieux, France; 8 High Energy Physics Division, Argonne National Laboratory, Argonne, IL USA; 9 Department of Physics, University of Arizona, Tucson, AZ USA; 10 Department of Physics, The University of Texas at Arlington, Arlington, TX USA; 11 Physics Department, National and Kapodistrian University of Athens, Athens, Greece; 12 Physics Department, National Technical University of Athens, Zografou, Greece; 13 Department of Physics, The University of Texas at Austin, Austin, TX USA; 14 Institute of Physics, Azerbaijan Academy of Sciences, Baku, Azerbaijan; 15 Institut de Física d’Altes Energies (IFAE), The Barcelona Institute of Science and Technology, Barcelona, Spain; 16 Institute of Physics, University of Belgrade, Belgrade, Serbia; 17 Department for Physics and Technology, University of Bergen, Bergen, Norway; 18 Physics Division, Lawrence Berkeley National Laboratory and University of California, Berkeley, CA USA; 19 Department of Physics, Humboldt University, Berlin, Germany; 20 Albert Einstein Center for Fundamental Physics and Laboratory for High Energy Physics, University of Bern, Bern, Switzerland; 21 School of Physics and Astronomy, University of Birmingham, Birmingham, UK; 22 Department of Physics, Bogazici University, Istanbul, Turkey; 23 Department of Physics Engineering, Gaziantep University, Gaziantep, Turkey; 24 Faculty of Engineering and Natural Sciences, Istanbul Bilgi University, Istanbul, Turkey; 25 Faculty of Engineering and Natural Sciences, Bahcesehir University, Istanbul, Turkey; 26 Centro de Investigaciones, Universidad Antonio Narino, Bogotá, Colombia; 27 INFN Sezione di Bologna, Bologna, Italy; 28 Dipartimento di Fisica e Astronomia, Università di Bologna, Bologna, Italy; 29 Physikalisches Institut, University of Bonn, Bonn, Germany; 30 Department of Physics, Boston University, Boston, MA USA; 31 Department of Physics, Brandeis University, Waltham, MA USA; 32 Universidade Federal do Rio De Janeiro COPPE/EE/IF, Rio de Janeiro, Brazil; 33 Electrical Circuits Department, Federal University of Juiz de Fora (UFJF), Juiz de Fora, Brazil; 34 Federal University of Sao Joao del Rei (UFSJ), Sao Joao del Rei, Brazil; 35 Instituto de Fisica, Universidade de Sao Paulo, Sao Paulo, Brazil; 36 Physics Department, Brookhaven National Laboratory, Upton, NY USA; 37 Transilvania University of Brasov, Brasov, Romania; 38 Horia Hulubei National Institute of Physics and Nuclear Engineering, Bucharest, Romania; 39 Department of Physics, Alexandru Ioan Cuza University of Iasi, Iasi, Romania; 40 Physics Department, National Institute for Research and Development of Isotopic and Molecular Technologies, Cluj Napoca, Romania; 41 University Politehnica Bucharest, Bucharest, Romania; 42 West University in Timisoara, Timisoara, Romania; 43 Departamento de Física, Universidad de Buenos Aires, Buenos Aires, Argentina; 44 Cavendish Laboratory, University of Cambridge, Cambridge, UK; 45 Department of Physics, Carleton University, Ottawa, ON Canada; 46 CERN, Geneva, Switzerland; 47 Enrico Fermi Institute, University of Chicago, Chicago, IL USA; 48 Departamento de Física, Pontificia Universidad Católica de Chile, Santiago, Chile; 49 Departamento de Física, Universidad Técnica Federico Santa María, Valparaiso, Chile; 50 Institute of High Energy Physics, University of CAS, Chinese Academy of Sciences, Beijing, China; 51 Department of Physics, Nanjing University, Nanjing, Jiangsu China; 52 Physics Department, Tsinghua University, Beijing, 100084 China; 53 Department of Modern Physics and State Key Laboratory of Particle Detection and Electronics, University of Science and Technology of China, Hefei, Anhui China; 54 School of Physics, Shandong University, Jinan, Shandong China; 55 Department of Physics and Astronomy, Key Laboratory for Particle Physics, Astrophysics and Cosmology, Ministry of Education, Shanghai Key Laboratory for Particle Physics and Cosmology, Shanghai Jiao Tong University, Shanghai (also at PKU-CHEP), Shanghai, China; 56 Université Clermont Auvergne, CNRS/IN2P3, LPC, Clermont-Ferrand, France; 57 Nevis Laboratory, Columbia University, Irvington, NY USA; 58 Niels Bohr Institute, University of Copenhagen, Copenhagen, Denmark; 59 INFN Gruppo Collegato di Cosenza, Laboratori Nazionali di Frascati, Frascati, Italy; 60 Dipartimento di Fisica, Università della Calabria, Rende, Italy; 61 Faculty of Physics and Applied Computer Science, AGH University of Science and Technology, Kraków, Poland; 62 Marian Smoluchowski Institute of Physics, Jagiellonian University, Kraków, Poland; 63 Institute of Nuclear Physics, Polish Academy of Sciences, Kraków, Poland; 64 Physics Department, Southern Methodist University, Dallas, TX USA; 65 Physics Department, University of Texas at Dallas, Richardson, TX USA; 66 DESY, Hamburg, Zeuthen, Germany; 67 Lehrstuhl für Experimentelle Physik IV, Technische Universität Dortmund, Dortmund, Germany; 68 Institut für Kern- und Teilchenphysik, Technische Universität Dresden, Dresden, Germany; 69 Department of Physics, Duke University, Durham, NC USA; 70 SUPA-School of Physics and Astronomy, University of Edinburgh, Edinburgh, UK; 71 INFN Laboratori Nazionali di Frascati, Frascati, Italy; 72 Fakultät für Mathematik und Physik, Albert-Ludwigs-Universität, Freiburg, Germany; 73 Departement de Physique Nucleaire et Corpusculaire, Université de Genève, Geneva, Switzerland; 74 INFN Sezione di Genova, Genoa, Italy; 75 Dipartimento di Fisica, Università di Genova, Genoa, Italy; 76 E. Andronikashvili Institute of Physics, Iv. Javakhishvili Tbilisi State University, Tbilisi, Georgia; 77 High Energy Physics Institute, Tbilisi State University, Tbilisi, Georgia; 78 II Physikalisches Institut, Justus-Liebig-Universität Giessen, Giessen, Germany; 79 SUPA-School of Physics and Astronomy, University of Glasgow, Glasgow, UK; 80 II Physikalisches Institut, Georg-August-Universität, Göttingen, Germany; 81 Laboratoire de Physique Subatomique et de Cosmologie, Université Grenoble-Alpes, CNRS/IN2P3, Grenoble, France; 82 Laboratory for Particle Physics and Cosmology, Harvard University, Cambridge, MA USA; 83 Kirchhoff-Institut für Physik, Ruprecht-Karls-Universität Heidelberg, Heidelberg, Germany; 84 Physikalisches Institut, Ruprecht-Karls-Universität Heidelberg, Heidelberg, Germany; 85 ZITI Institut für technische Informatik, Ruprecht-Karls-Universität Heidelberg, Mannheim, Germany; 86 Faculty of Applied Information Science, Hiroshima Institute of Technology, Hiroshima, Japan; 87 Department of Physics, The Chinese University of Hong Kong, Shatin, N.T. Hong Kong; 88 Department of Physics, The University of Hong Kong, Hong Kong, China; 89 Department of Physics and Institute for Advanced Study, The Hong Kong University of Science and Technology, Clear Water Bay, Kowloon, Hong Kong, China; 90 Department of Physics, National Tsing Hua University, Hsinchu City, Taiwan; 91 Department of Physics, Indiana University, Bloomington, IN USA; 92 Institut für Astro- und Teilchenphysik, Leopold-Franzens-Universität, Innsbruck, Austria; 93 University of Iowa, Iowa City, IA USA; 94 Department of Physics and Astronomy, Iowa State University, Ames, IA USA; 95 Joint Institute for Nuclear Research, JINR Dubna, Dubna, Russia; 96 KEK, High Energy Accelerator Research Organization, Tsukuba, Japan; 97 Graduate School of Science, Kobe University, Kobe, Japan; 98 Faculty of Science, Kyoto University, Kyoto, Japan; 99 Kyoto University of Education, Kyoto, Japan; 100 Research Center for Advanced Particle Physics and Department of Physics, Kyushu University, Fukuoka, Japan; 101 Instituto de Física La Plata, Universidad Nacional de La Plata and CONICET, La Plata, Argentina; 102 Physics Department, Lancaster University, Lancaster, UK; 103 INFN Sezione di Lecce, Lecce, Italy; 104 Dipartimento di Matematica e Fisica, Università del Salento, Lecce, Italy; 105 Oliver Lodge Laboratory, University of Liverpool, Liverpool, UK; 106 Department of Experimental Particle Physics, Jožef Stefan Institute and Department of Physics, University of Ljubljana, Ljubljana, Slovenia; 107 School of Physics and Astronomy, Queen Mary University of London, London, UK; 108 Department of Physics, Royal Holloway University of London, Surrey, UK; 109 Department of Physics and Astronomy, University College London, London, UK; 110 Louisiana Tech University, Ruston, LA USA; 111 Laboratoire de Physique Nucléaire et de Hautes Energies, UPMC and Université Paris-Diderot and CNRS/IN2P3, Paris, France; 112 Fysiska Institutionen, Lunds Universitet, Lund, Sweden; 113 Departamento de Fisica Teorica C-15, Universidad Autonoma de Madrid, Madrid, Spain; 114 Institut für Physik, Universität Mainz, Mainz, Germany; 115 School of Physics and Astronomy, University of Manchester, Manchester, UK; 116 CPPM, Aix-Marseille Université and CNRS/IN2P3, Marseille, France; 117 Department of Physics, University of Massachusetts, Amherst, MA USA; 118 Department of Physics, McGill University, Montreal, QC Canada; 119 School of Physics, University of Melbourne, Victoria, Australia; 120 Department of Physics, The University of Michigan, Ann Arbor, MI USA; 121 Department of Physics and Astronomy, Michigan State University, East Lansing, MI USA; 122 INFN Sezione di Milano, Milan, Italy; 123 Dipartimento di Fisica, Università di Milano, Milan, Italy; 124 B.I. Stepanov Institute of Physics, National Academy of Sciences of Belarus, Minsk, Republic of Belarus; 125 Research Institute for Nuclear Problems of Byelorussian State University, Minsk, Republic of Belarus; 126 Group of Particle Physics, University of Montreal, Montreal, QC Canada; 127 P.N. Lebedev Physical Institute of the Russian Academy of Sciences, Moscow, Russia; 128 Institute for Theoretical and Experimental Physics (ITEP), Moscow, Russia; 129 National Research Nuclear University MEPhI, Moscow, Russia; 130 D.V. Skobeltsyn Institute of Nuclear Physics, M.V. Lomonosov Moscow State University, Moscow, Russia; 131 Fakultät für Physik, Ludwig-Maximilians-Universität München, Munich, Germany; 132 Max-Planck-Institut für Physik (Werner-Heisenberg-Institut), Munich, Germany; 133 Nagasaki Institute of Applied Science, Nagasaki, Japan; 134 Graduate School of Science and Kobayashi-Maskawa Institute, Nagoya University, Nagoya, Japan; 135 INFN Sezione di Napoli, Naples, Italy; 136 Dipartimento di Fisica, Università di Napoli, Naples, Italy; 137 Department of Physics and Astronomy, University of New Mexico, Albuquerque, NM USA; 138 Institute for Mathematics, Astrophysics and Particle Physics, Radboud University Nijmegen/Nikhef, Nijmegen, The Netherlands; 139 Nikhef National Institute for Subatomic Physics and University of Amsterdam, Amsterdam, The Netherlands; 140 Department of Physics, Northern Illinois University, DeKalb, IL USA; 141 Budker Institute of Nuclear Physics, SB RAS, Novosibirsk, Russia; 142 Department of Physics, New York University, New York, NY USA; 143 Ohio State University, Columbus, OH USA; 144 Faculty of Science, Okayama University, Okayama, Japan; 145 Homer L. Dodge Department of Physics and Astronomy, University of Oklahoma, Norman, OK USA; 146 Department of Physics, Oklahoma State University, Stillwater, OK USA; 147 Palacký University, RCPTM, Olomouc, Czech Republic; 148 Center for High Energy Physics, University of Oregon, Eugene, OR USA; 149 LAL, Univ. Paris-Sud, CNRS/IN2P3, Université Paris-Saclay, Orsay, France; 150 Graduate School of Science, Osaka University, Osaka, Japan; 151 Department of Physics, University of Oslo, Oslo, Norway; 152 Department of Physics, Oxford University, Oxford, UK; 153 INFN Sezione di Pavia, Pavia, Italy; 154 Dipartimento di Fisica, Università di Pavia, Pavia, Italy; 155 Department of Physics, University of Pennsylvania, Philadelphia, PA USA; 156 National Research Centre “Kurchatov Institute” B.P. Konstantinov Petersburg Nuclear Physics Institute, St. Petersburg, Russia; 157 INFN Sezione di Pisa, Pisa, Italy; 158 Dipartimento di Fisica E. Fermi, Università di Pisa, Pisa, Italy; 159 Department of Physics and Astronomy, University of Pittsburgh, Pittsburgh, PA USA; 160 Laboratório de Instrumentação e Física Experimental de Partículas-LIP, Lisbon, Portugal; 161 Faculdade de Ciências, Universidade de Lisboa, Lisbon, Portugal; 162 Department of Physics, University of Coimbra, Coimbra, Portugal; 163 Centro de Física Nuclear da Universidade de Lisboa, Lisbon, Portugal; 164 Departamento de Fisica, Universidade do Minho, Braga, Portugal; 165 Departamento de Fisica Teorica y del Cosmos and CAFPE, Universidad de Granada, Granada, Spain; 166 Dep Fisica and CEFITEC of Faculdade de Ciencias e Tecnologia, Universidade Nova de Lisboa, Caparica, Lisbon, Portugal; 167 Institute of Physics, Academy of Sciences of the Czech Republic, Prague, Czech Republic; 168 Czech Technical University in Prague, Prague, Czech Republic; 169 Faculty of Mathematics and Physics, Charles University, Prague, Czech Republic; 170 State Research Center Institute for High Energy Physics (Protvino), NRC KI, Protvino, Russia; 171 Particle Physics Department, Rutherford Appleton Laboratory, Didcot, UK; 172 INFN Sezione di Roma, Rome, Italy; 173 Dipartimento di Fisica, Sapienza Università di Roma, Rome, Italy; 174 INFN Sezione di Roma Tor Vergata, Rome, Italy; 175 Dipartimento di Fisica, Università di Roma Tor Vergata, Rome, Italy; 176 INFN Sezione di Roma Tre, Rome, Italy; 177 Dipartimento di Matematica e Fisica, Università Roma Tre, Rome, Italy; 178 Faculté des Sciences Ain Chock, Réseau Universitaire de Physique des Hautes Energies-Université Hassan II, Casablanca, Morocco; 179 Centre National de l’Energie des Sciences Techniques Nucleaires, Rabat, Morocco; 180 Faculté des Sciences Semlalia, Université Cadi Ayyad, LPHEA-Marrakech, Marrakech, Morocco; 181 Faculté des Sciences, Université Mohamed Premier and LPTPM, Oujda, Morocco; 182 Faculté des Sciences, Université Mohammed V, Rabat, Morocco; 183 DSM/IRFU (Institut de Recherches sur les Lois Fondamentales de l’Univers), CEA Saclay (Commissariat à l’Energie Atomique et aux Energies Alternatives), Gif-sur-Yvette, France; 184 Santa Cruz Institute for Particle Physics, University of California Santa Cruz, Santa Cruz, CA USA; 185 Department of Physics, University of Washington, Seattle, WA USA; 186 Department of Physics and Astronomy, University of Sheffield, Sheffield, UK; 187 Department of Physics, Shinshu University, Nagano, Japan; 188 Department Physik, Universität Siegen, Siegen, Germany; 189 Department of Physics, Simon Fraser University, Burnaby, BC Canada; 190 SLAC National Accelerator Laboratory, Stanford, CA USA; 191 Faculty of Mathematics, Physics and Informatics, Comenius University, Bratislava, Slovak Republic; 192 Department of Subnuclear Physics, Institute of Experimental Physics of the Slovak Academy of Sciences, Kosice, Slovak Republic; 193 Department of Physics, University of Cape Town, Cape Town, South Africa; 194 Department of Physics, University of Johannesburg, Johannesburg, South Africa; 195 School of Physics, University of the Witwatersrand, Johannesburg, South Africa; 196 Department of Physics, Stockholm University, Stockholm, Sweden; 197 The Oskar Klein Centre, Stockholm, Sweden; 198 Physics Department, Royal Institute of Technology, Stockholm, Sweden; 199 Departments of Physics and Astronomy and Chemistry, Stony Brook University, Stony Brook, NY USA; 200 Department of Physics and Astronomy, University of Sussex, Brighton, UK; 201 School of Physics, University of Sydney, Sydney, Australia; 202 Institute of Physics, Academia Sinica, Taipei, Taiwan; 203 Department of Physics, Technion: Israel Institute of Technology, Haifa, Israel; 204 Raymond and Beverly Sackler School of Physics and Astronomy, Tel Aviv University, Tel Aviv, Israel; 205 Department of Physics, Aristotle University of Thessaloniki, Thessaloníki, Greece; 206 International Center for Elementary Particle Physics and Department of Physics, The University of Tokyo, Tokyo, Japan; 207 Graduate School of Science and Technology, Tokyo Metropolitan University, Tokyo, Japan; 208 Department of Physics, Tokyo Institute of Technology, Tokyo, Japan; 209 Tomsk State University, Tomsk, Russia; 210 Department of Physics, University of Toronto, Toronto, ON Canada; 211 INFN-TIFPA, Trento, Italy; 212 University of Trento, Trento, Italy; 213 TRIUMF, Vancouver, BC Canada; 214 Department of Physics and Astronomy, York University, Toronto, ON Canada; 215 Faculty of Pure and Applied Sciences, and Center for Integrated Research in Fundamental Science and Engineering, University of Tsukuba, Tsukuba, Japan; 216 Department of Physics and Astronomy, Tufts University, Medford, MA USA; 217 Department of Physics and Astronomy, University of California Irvine, Irvine, CA USA; 218 INFN Gruppo Collegato di Udine, Sezione di Trieste, Udine, Italy; 219 ICTP, Trieste, Italy; 220 Dipartimento di Chimica, Fisica e Ambiente, Università di Udine, Udine, Italy; 221 Department of Physics and Astronomy, University of Uppsala, Uppsala, Sweden; 222 Department of Physics, University of Illinois, Urbana, IL USA; 223 Instituto de Fisica Corpuscular (IFIC), Centro Mixto Universidad de Valencia - CSIC, Valencia, Spain; 224 Department of Physics, University of British Columbia, Vancouver, BC Canada; 225 Department of Physics and Astronomy, University of Victoria, Victoria, BC Canada; 226 Department of Physics, University of Warwick, Coventry, UK; 227 Waseda University, Tokyo, Japan; 228 Department of Particle Physics, The Weizmann Institute of Science, Rehovot, Israel; 229 Department of Physics, University of Wisconsin, Madison, WI USA; 230 Fakultät für Physik und Astronomie, Julius-Maximilians-Universität, Würzburg, Germany; 231 Fakultät für Mathematik und Naturwissenschaften, Fachgruppe Physik, Bergische Universität Wuppertal, Wuppertal, Germany; 232 Department of Physics, Yale University, New Haven, CT USA; 233 Yerevan Physics Institute, Yerevan, Armenia; 234 CH-1211, Geneva 23, Switzerland; 235 Centre de Calcul de l’Institut National de Physique Nucléaire et de Physique des Particules (IN2P3), Villeurbanne, France; 236 Academia Sinica Grid Computing, Institute of Physics, Academia Sinica, Taipei, Taiwan; 237 CERN, 1211 Geneva 23, Switzerland

## Abstract

The rejection of forward jets originating from additional proton–proton interactions (pile-up) is crucial for a variety of physics analyses at the LHC, including Standard Model measurements and searches for physics beyond the Standard Model. The identification of such jets is challenging due to the lack of track and vertex information in the pseudorapidity range $|\eta |>2.5$. This paper presents a novel strategy for forward pile-up jet tagging that exploits jet shapes and topological jet correlations in pile-up interactions. Measurements of the per-jet tagging efficiency are presented using a data set of 3.2 fb$^{-1}$ of proton–proton collisions at a centre-of-mass energy of 13 $\,\text {TeV}$ collected with the ATLAS detector. The fraction of pile-up jets rejected in the range $2.5<|\eta |<4.5$ is estimated in simulated events with an average of 22 interactions per bunch-crossing. It increases with jet transverse momentum and, for jets with transverse momentum between 20 and 50 GeV, it ranges between 49% and 67% with an efficiency of 85% for selecting hard-scatter jets. A case study is performed in Higgs boson production via the vector-boson fusion process, showing that these techniques mitigate the background growth due to additional proton–proton interactions, thus enhancing the reach for such signatures.

## Introduction

In order to enhance the capability of the experiments to discover physics beyond the Standard Model, the Large Hadron Collider (LHC) operates at the conditions yielding the highest integrated luminosity achievable. Therefore, the collisions of proton bunches result not only in large transverse-momentum transfer proton–proton ($pp$) interactions, but also in additional collisions within the same bunch crossing, primarily consisting of low-energy quantum chromodynamics (QCD) processes. Such additional $pp$ collisions are referred to as *in-time* *pile-up* interactions. In addition to in-time pile-up, *out-of-time* pile-up refers to the energy deposits in the ATLAS calorimeter from previous and following bunch crossings with respect to the triggered event. In this paper, in-time and out-of-time pile-up are referred collectively as pile-up (PU).

**Fig. 1:**
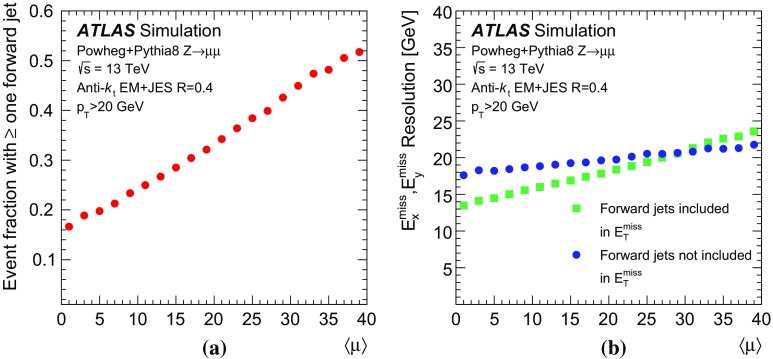
**a** Fraction of simulated $Z +$jets events with at least one forward jet and **b** the resolution of the $E_{\text {T}}^{\text {miss}}$ components $E_x^{\text {miss}}$ and $E_y^{\text {miss}}$ as a function of $\langle \mu \rangle$. Jets and $E_{\text {T}}^{\text {miss}}$ definitions are described in Sect. 2

In Ref. [1] it was shown that pile-up jets can be effectively removed using track and vertex information with the jet-vertex-tagger ($\mathrm {JVT}$) technique. The CMS Collaboration employs a pile-up mitigation strategy based on tracks and jet shapes [2]. A limitation of the $\mathrm {JVT}$ discriminant used by the ATLAS Collaboration is that it can only be used for jets within the coverage^1^ of the tracking detector, $|\eta |<2.5$. However, in the ATLAS detector, jets are reconstructed in the range $|\eta |<4.5$. The rejection of pile-up jets in the *forward* region, here defined as $2.5<|\eta |<4.5$, is crucial to enhance the sensitivity of key analyses such as the measurement of Higgs boson production in the vector-boson fusion (VBF) process. Figure 1a shows how the fraction of $Z +$jets events with at least one forward jet^2^ with $p_{\text {T}} >20\,\text {GeV}$, an important background for VBF analyses, rises quickly with busier pile-up conditions, quantified by the average number of interactions per bunch crossing ($\langle \mu \rangle$). Likewise, the resolution of the missing transverse momentum ($E_{\text {T}}^{\text {miss}}$) components $E_x^{\text {miss}}$ and $E_y^{\text {miss}}$ in $Z +$jets events is also affected by the presence of forward pile-up jets. The inclusion of forward jets allows a more precise $E_{\text {T}}^{\text {miss}}$ calculation but a more pronounced pile-up dependence, as shown in Fig. 1b. At higher $\langle \mu \rangle$, improving the $E_{\text {T}}^{\text {miss}}$ resolution depends on rejecting all forward jets, unless the impact of pile-up jets specifically can be mitigated.

In this paper, the phenomenology of pile-up jets with $|\eta |>2.5$ is investigated in detail, and techniques to identify and reject them are presented. The paper is organized as follows. Section 2 briefly describes the ATLAS detector, the event reconstruction and selection. The physical origin and classification of pile-up jets are described in Sect. 3. Section 4 describes the use of jet shape variables for the identification and rejection of forward pile-up jets. The *forward* $\mathrm {JVT}$ ($\mathrm {fJVT}$) technique is presented in Sect. 5 along with its performance and efficiency measurements. The usage of jet shape variables in improving $\mathrm {fJVT}$ performance is presented in Sect. 6, while the application of forward pile-up jet rejection in a VBF analysis is discussed in Sect. 7. The conclusions are presented in Sect. 8.

## Experimental setup

### ATLAS detector

The ATLAS detector is a general-purpose particle detector covering almost $4\pi$ in solid angle and consisting of a tracking system called the inner detector (ID), a calorimeter system, and a muon spectrometer (MS). The details of the detector are given in Refs. [3–5].

The ID consists of silicon pixel and microstrip tracking detectors covering the pseudorapidity range of $|\eta | < 2.5$ and a straw-tube tracker covering $|\eta | < 2.0$. These components are immersed in an axial 2 T magnetic field provided by a superconducting solenoid.

The electromagnetic (EM) and hadronic calorimeters are composed of multiple subdetectors covering the range $|\eta |<4.9$, generally divided into barrel ($|\eta | < 1.4$), endcap ($1.4< |\eta | < 3.2$) and forward ($3.2< |\eta | < 4.9$) regions. The barrel and endcap sections of the EM calorimeter use liquid argon (LAr) as the active medium and lead absorbers. The hadronic endcap calorimeter ($1.5<|\eta |<3.2$) uses copper absorbers and LAr, while in the forward ($3.1<|\eta |<4.9$) region LAr, copper and tungsten are used. The LAr calorimeter read-out [6], with a pulse length between 60 and 600 ns, is sensitive to signals from the preceding 24 bunch crossings. It uses bipolar shaping with positive and negative output, which ensures that the signal induced by out-of-time pile-up averages to zero. In the region $|\eta |<1.7$, the hadronic (Tile) calorimeter is constructed from steel absorber and scintillator tiles and is separated into barrel ($|\eta |<1.0$) and extended barrel ($0.8<|\eta |<1.7$) sections. The fast response of the Tile calorimeter makes it less sensitive to out-of-time pile-up.

The MS forms the outer layer of the ATLAS detector and is dedicated to the detection and measurement of high-energy muons in the region $|\eta |<2.7$. A multi-level trigger system of dedicated hardware and software filters is used to select $pp$ collisions producing high-$p_{\text {T}}$ particles.

### Data and MC samples

The studies presented in this paper are performed using a data set of *pp* collisions at $\sqrt{s}=13\,\text {TeV}$, corresponding to an integrated luminosity of 3.2 fb$^{-1}$, collected in 2015 during which the LHC operated with a bunch spacing of 25 ns. There are on average 13.5 interactions per bunch crossing in the data sample used for the analysis.

Samples of simulated events used for comparisons with data are reweighted to match the distribution of the number of pile-up interactions observed in data. The average number of interactions per bunch crossing $\langle \mu \rangle$ in the data used as reference for the reweighting is divided by a scale factor of $1.16\pm 0.07$. This scale factor takes into account the fraction of visible cross-section due to inelastic $pp$ collisions as measured in the data [7] and is required to obtain good agreement with the number of inelastic interactions reconstructed in the tracking detector as predicted in the reweighted simulation. In order to extend the study of the pile-up dependence, simulated samples with an average of 22 interactions per bunch crossing are also used. Dijet events are simulated with the Pythia8.186  [8] event generator using the NNPDF2.3LO [9] set of parton distribution functions (PDFs) and the parameter values set according to the A14 underlying-event tune [10]. Simulated ${t\bar{t}}$ events are generated with powheg box  v2.0 [11–13] using the CT10 PDF set [14]; Pythia6.428  [15] is used for fragmentation and hadronization with the Perugia2012 [16] tune that employs the CTEQ6L1 [17] PDF set. A sample of leptonically decaying Z bosons produced with jets ($Z (\rightarrow \ell \ell )$+jets) and VBF $H\rightarrow \tau \tau$ samples are generated with powheg box v1.0 and Pythia8.186 is used for fragmentation and hadronization with the AZNLO tune [18] and the CTEQ6L1 PDF set. For all samples, the EvtGen v1.2.0 program [19] is used for properties of the bottom and charm hadron decays. The effect of in-time as well as out-of-time pile-up is simulated using minimum-bias events generated with Pythia8.186 to reflect the pile-up conditions during the 2015 data-taking period, using the A2 tune [20] and the MSTW2008LO [21] PDF set. All generated events are processed with a detailed simulation of the ATLAS detector response [22] based on Geant4  [23] and subsequently reconstructed and analysed in the same way as the data.

### Event reconstruction

The raw data collected by the ATLAS detector is reconstructed in the form of particle candidates and jets using various pattern recognition algorithms. The reconstruction used in this analysis are detailed in Ref. [1], while an overview is presented in this section.

**Calorimeter clusters and towers**

Jets in ATLAS are reconstructed from clusters of energy deposits in the calorimeters. Two methods of combining calorimeter cell information are considered in this paper: topological clusters and towers.

Topological clusters (topo-clusters) [24] are built from neighbouring calorimeter cells. The algorithm uses as seeds calorimeter cells with energy significance^3^ $|E_\mathrm {cell}|/\sigma _\mathrm {noise}>4$, combines all neighbouring cells with $|E_\mathrm {cell}|/\sigma _\mathrm {noise}>2$ and finally adds neighbouring cells without any significance requirement. Topo-clusters are used as input for jet reconstruction.

Calorimeter towers are fixed-size objects ($\Delta \eta \times \Delta \phi =0.1\times 0.1$) [26] that ensure a uniform segmentation of the calorimeter information. Instead of building clusters, the cells are projected onto a fixed grid in $\eta$ and $\phi$ corresponding to 6400 towers. Calorimeter cells which completely fit within a tower contribute their total energy to the single tower. Other cells extending beyond the tower boundary contribute to multiple towers, depending on the overlap fraction of the cell area with the towers. In the following, towers are matched geometrically to jets reconstructed using topo-clusters and are used for jet classification.

**Vertices and tracks**

The event hard-scatter primary vertex is defined as the reconstructed primary vertex with the largest $\sum p_\mathrm {T}^2$ of constituent tracks. When evaluating performance in simulation, only events where the reconstructed hard-scatter primary vertex lies $|\Delta z|<0.1$ mm from the true hard-scatter interaction are considered. For the physics processes considered, the reconstructed hard-scatter primary vertex matches the true hard-scatter interaction more than 95% of the time. Tracks are required to have $p_{\text {T}} > 0.5\,\text {GeV}$ and to satisfy quality criteria designed to reject poorly measured or fake tracks [27]. Tracks are assigned to primary vertices based on the track-to-vertex matching resulting from the vertex reconstruction. Tracks not included in vertex reconstruction are assigned to the nearest vertex based on the distance $|\Delta z \times \sin \theta |$, up to a maximum distance of 3.0 mm. Tracks not matched to any vertex are not considered. Tracks are then assigned to jets by adding them to the jet clustering process with infinitesimal $p_{\text {T}}$ , a procedure known as ghost-association [28].

**Jets**

Jets are reconstructed from topo-clusters at the EM scale^4^ using the anti-$k_t$ [29] algorithm, as implemented in Fastjet 2.4.3  [30], with a radius parameter $R=0.4$. After a jet-area-based subtraction of pile-up energy, a response correction is applied to each jet reconstructed in the calorimeter to calibrate it to the particle-level jet energy scale [1, 25, 31]. Unless noted otherwise, jets are required to have $20\,\text {GeV}< p_{\text {T}} < 50\,\text {GeV}$. Higher-$p_{\text {T}}$ forward jets are ignored due to their negligible pile-up rate at the pile-up conditions considered in this paper. *Central* jets are required to be within $|\eta |$ of 2.5 so that most of their charged particles are within the tracking coverage of the inner detector. *Forward* jets are those in the region $2.5<|\eta |<4.5$, and no tracks associated with their charged particles are measured beyond $|\eta |=2.5$.

Jets built from particles in the Monte Carlo generator’s event record (“truth particles”) are also considered. Truth-particle jets are reconstructed using the anti-$k_t$ algorithm with $R=0.4$ from stable^5^ final-state truth particles from the simulated hard-scatter (*truth-particle hard-scatter jets*) or in-time pile-up (*truth-particle pile-up jets*) interaction of choice. A third type of truth-particle jet (*inclusive truth-particle jets*) is reconstructed by considering truth particles from all interactions simultaneously, in order to study the effects of pile-up interactions on truth-particle pile-up jets.

The simulation studies in this paper require a classification of the reconstructed jets into three categories: *hard-scatter jets*, *QCD pile-up jets*, and *stochastic pile-up jets*. Jets are thus truth-labelled based on a matching criterion to truth-particle jets. Similarly to Ref. [1], jets are first classified as hard-scatter or pile-up jets. Jets are labelled as hard-scatter jets if a truth-particle hard-scatter jet with $p_{\text {T}} > 10\,\text {GeV}$ is found within $\Delta R = \sqrt{(\Delta \eta )^2 + (\Delta \phi )^2}$ of 0.3. The $p_{\text {T}} >10\,\text {GeV}$ requirement is used to avoid accidental matches of reconstructed jets with soft activity from the hard-scatter interaction. In cases where more than one truth-particle jet is matched, $p_{\text {T}} ^\mathrm {truth}$ is defined from the highest-$p_{\text {T}}$ truth-particle hard-scatter jet within $\Delta R$ of 0.3.

Jets are labelled as pile-up jets if no truth-particle hard-scatter jet with $p_{\text {T}} > 4\,\text {GeV}$ is found within $\Delta R$ of 0.6. These pile-up jets are further classified as QCD pile-up if they are matched within $\Delta R<0.3$ to a truth-particle pile-up jet or as stochastic pile-up jets if there is no truth-particle pile-up jet within $\Delta R<0.6$, requiring that truth-particle pile-up jets have $p_{\text {T}} > 10\,\text {GeV}$ in both cases. Jets with $0.3<\Delta R<0.6$ relative to truth-particle hard-scatter jets with $p_{\text {T}} > 10\,\text {GeV}$ or $\Delta R<0.3$ of truth-particle hard-scatter jets with $4\,\text {GeV}< p_{\text {T}} < 10\,\text {GeV}$ are not labelled because their nature cannot be unambiguously determined. These jets are therefore not used for performance based on simulation.

**Jet Vertex Tagger**

The Jet Vertex Tagger (JVT) is built out of the combination of two jet variables, $\mathrm {corrJVF}$ and $R_\mathrm {pT} ^0$, that provide information to separate hard-scatter jets from pile-up jets. The quantity $\mathrm {corrJVF}$  [1] is defined for each jet as

1 $$\begin{aligned} \mathrm {corrJVF} = \frac{\sum {p_{\text {T}} ^{\mathrm {trk}}(\mathrm {PV}_0)}}{\sum p_{\text {T}} ^{\mathrm {trk}}(\mathrm {PV}_0) + \frac{p_{\text {T}} ^{\mathrm {PU}}}{ (k \cdot n_\mathrm {trk}^\mathrm {PU})}}, \end{aligned}$$

where PV$_i$ denotes the reconstructed event vertices (PV$_0$ is the identified hard-scatter vertex and the PV$_i$ are sorted by decreasing $\sum p_\mathrm {T}^2$), and $\sum {p_{\text {T}} ^{\mathrm {trk}}(\mathrm {PV}_0)}$ is the scalar $p_{\text {T}}$ sum of the tracks that are associated with the jet and originate from the hard-scatter vertex. The term $p_{\text {T}} ^{\mathrm {PU}}=\sum _{i\ge 1}\sum p_{\text {T}} ^{\mathrm {trk}}(\mathrm {PV}_i)$ denotes the scalar $p_{\text {T}}$ sum of the tracks associated with the jet and originating from pile-up vertices. To correct for the linear increase of $p_{\text {T}} ^{\mathrm {PU}}$ with the total number of pile-up tracks per event ($n_\mathrm {trk}^\mathrm {PU}$), $p_{\text {T}} ^{\mathrm {PU}}$ is divided by $(k \cdot n_\mathrm {trk}^\mathrm {PU})$ with the parameter *k* set to 0.01 [1].^6^

The variable $R_\mathrm {pT} ^0$ is defined as the scalar $p_{\text {T}}$ sum of the tracks that are associated with the jet and originate from the hard-scatter vertex divided by the fully calibrated jet $p_{\text {T}}$, which includes pile-up subtraction:

2 $$\begin{aligned} R_\mathrm {pT} ^0 = \frac{\sum {p_{\text {T}} ^{\mathrm {trk}}(\mathrm {PV}_0)}}{p_{\text {T}} ^\mathrm {jet}}. \end{aligned}$$

This observable tests the compatibility between the jet $p_{\text {T}}$ and the total $p_{\text {T}}$ of the hard-scatter charged particles within the jet. Its average value for hard-scatter jets is approximately 0.5, as the numerator does not account for the neutral particles in the jet. The $\mathrm {JVT}$ discriminant is built by defining a two-dimensional likelihood based on a k-nearest neighbour (kNN) algorithm [32]. An extension of the $R_\mathrm {pT} ^0$ variable computed with respect to any vertex *i* in the event, $R_\mathrm {pT} ^i=\sum _k{p_{\text {T}} ^{\mathrm {trk}_k}(\mathrm {PV}_i)}/p_{\text {T}} ^\mathrm {jet}$, is also used in this analysis.

*Electrons and muons* Electrons are built from EM clusters and associated ID tracks. They are required to satisfy $|\eta |<2.47$ and $p_\mathrm {T}>10\,\text {GeV}$, as well as reconstruction quality and isolation criteria [33]. Muons are built from an ID track (for $|\eta |<2.5$) and an MS track. Muons are required to satisfy $p_\mathrm {T}>10\,\text {GeV}$ as well as reconstruction quality and isolation criteria [34]. Correction factors are applied to simulated events to account for mismodelling of lepton isolation, trigger efficiency, and quality selection variables.

$E_{\text {T}}^{\text {miss}}$ The missing transverse momentum, $\varvec{E}_{\text {T}}^{\text {miss}}$, corresponds to the negative vector sum of the transverse momenta of selected electron, photon, and muon candidates, as well as jets and tracks not used in reconstruction [35]. The scalar magnitude $E_{\text {T}}^{\text {miss}}$ represents the total transverse momentum imbalance in an event.

## Origin and structure of pile-up jets

**Fig. 2:**
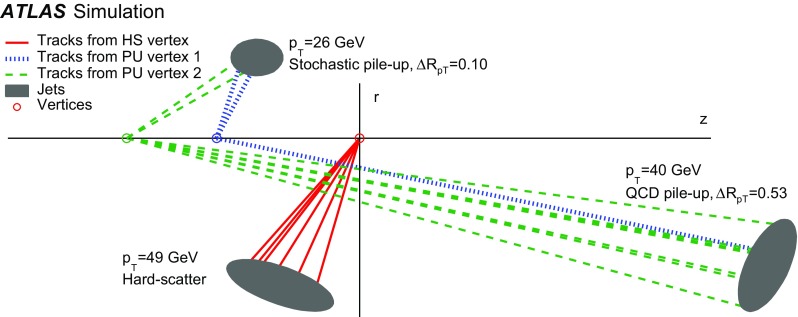
Display of a simulated event in *r*–*z* view containing a hard-scatter jet, a QCD pile-up jet, and a stochastic pile-up jet. The $\Delta R_\mathrm {pT}$ values (defined in Sect. 5.1) are quoted for the two pile-up jets

The additional transverse energy from pile-up interactions contributing to jets originating from the hard-scatter (HS) interaction is subtracted on an event-by-event basis using the jet-area method [1, 36]. However, the jet-area subtraction assumes a uniform pile-up distribution across the calorimeter, while local fluctuations of pile-up can cause additional jets to be reconstructed. The additional jets can be classified into two categories: *QCD pile-up jets*, where the particles in the jet stem mostly from a single QCD process occuring in a single pile-up interaction, and *stochastic jets*, which combine particles from different interactions. Figure 2 shows an event with a hard-scatter jet, a QCD pile-up jet and a stochastic pile-up jet. Most of the particles associated with the hard-scatter jet originate from the primary interaction. Most of the particles associated with the QCD pile-up jet originate from a single pile-up interaction. The stochastic pile-up jet includes particles associated with both pile-up interactions in the event, without a single prevalent source.

While this binary classification is convenient for the purpose of description, the boundary between the two categories is somewhat arbitrary. This is particularly true in harsh pile-up conditions, with dozens of concurrent *pp* interactions, where every jet, including those originating primarily from the identified hard-scatter interaction, also has contributions from multiple pile-up interactions.

In order to identify and reject forward pile-up jets, a twofold strategy is adopted. Stochastic jets have intrinsic differences in shape with respect to hard-scatter and QCD pile-up jets, and this shape can be used for discrimination. On the other hand, the calorimeter signature of QCD pile-up jets does not differ fundamentally from that of hard-scatter jets. Therefore, QCD pile-up jets are identified by exploiting transverse momentum conservation in individual pile-up interactions.

**Fig. 3:**
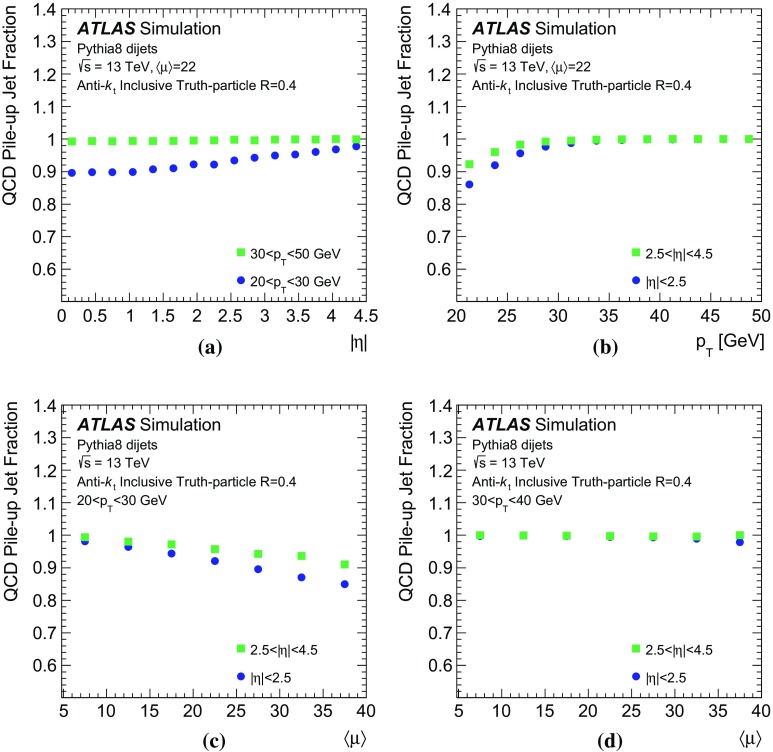
Fraction of pile-up tagged inclusive truth-particle jets classified as QCD pile-up jets as a function of **a** $|\eta |$, **b** $p_{\text {T}}$, and **c** $\langle \mu \rangle$ for jets with $20\,\text {GeV}<p_{\text {T}} <30\,\text {GeV}$ and **d** $30\,\text {GeV}<p_{\text {T}} <40\,\text {GeV}$, as estimated in dijet events with Pythia8.186 pile-up simulation. The inclusive truth-particle jets are reconstructed from truth particles originating from all in-time pile-up interactions

**Fig. 4:**
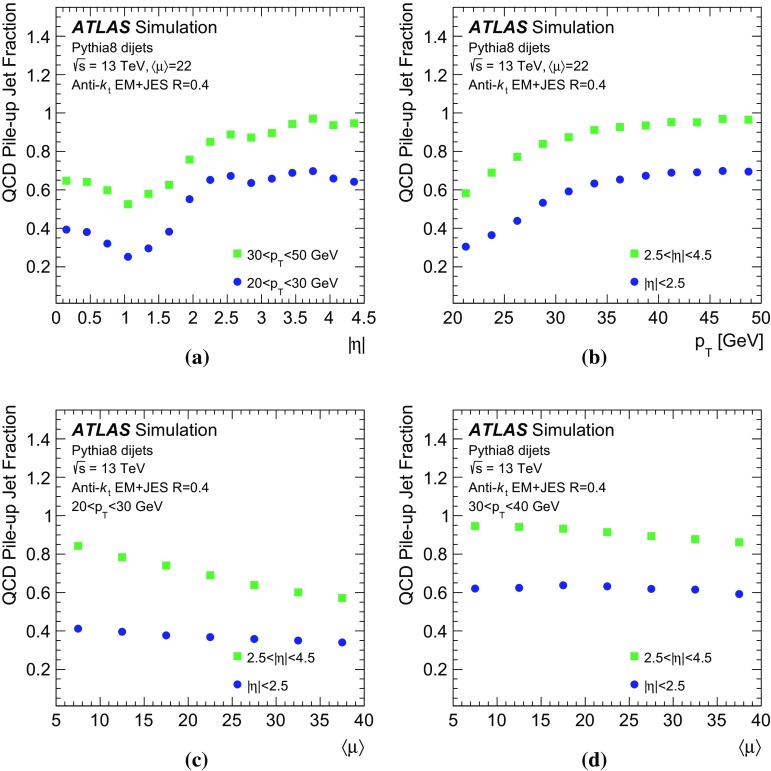
Fraction of reconstructed pile-up jets classified as QCD pile-up jets, as a function of **a** $|\eta |$, **b** $p_{\text {T}}$, and **c** $\langle \mu \rangle$ for jets with $20\,\text {GeV}<p_{\text {T}} <30\,\text {GeV}$ and **d** $30\,\text {GeV}<p_{\text {T}} <40\,\text {GeV}$, as estimated in dijet events with Pythia8.186 pile-up simulation

The nature of pile-up jets can vary significantly whether or not most of the jet energy originates from a single interaction. Figure 3 shows the fraction of QCD pile-up jets among all pile-up jets, when considering inclusive truth-particle jets. The corresponding distributions for reconstructed jets are shown in Fig. 4. When considering only in-time pile-up contributions (Fig. 3), the fraction of QCD pile-up jets depends on the pseudorapidity and $p_{\text {T}}$ of the jet and the average number of interactions per bunch crossing $\langle \mu \rangle$. Stochastic jets are more likely at low $p_{\text {T}}$ and $|\eta |$ and in harsher pile-up conditions. However, the comparison between Fig. 3, containing inclusive truth-particle jets, and Fig. 4, containing reconstructed jets, suggests that only a small fraction of stochastic jets are due to in-time pile-up. Indeed, the fraction of QCD pile-up jets decreases significantly once out-of-time pile-up effects and detector noise and resolution are taken into account. Even though the average amount of out-of-time energy is higher in the forward region, topo-clustering results in a stronger suppression of this contribution in the forward region. Therefore, the fraction of QCD pile-up jets increases in the forward region, and it constitutes more than 80% of pile-up jets with $p_{\text {T}}$ > 30 $\text {GeV}$overall. Similarly, the minimum at around $|\eta |=1$ corresponds to a maximum in the pile-up noise distribution [24], which results in a larger number of stochastic pile-up jets relative to QCD pile-up jets. The fraction of stochastic jets becomes more prominent at low $p_{\text {T}}$ and it grows as the number of interactions increases. The majority of pile-up jets in the forward region are QCD pile-up jets, although a sizeable fraction of stochastic jets is present in both the central and forward regions.

In the following, each source of forward pile-up jets is addressed with algorithms targeting its specific features.

## Stochastic pile-up jet tagging with time and shape information

**Fig. 5:**
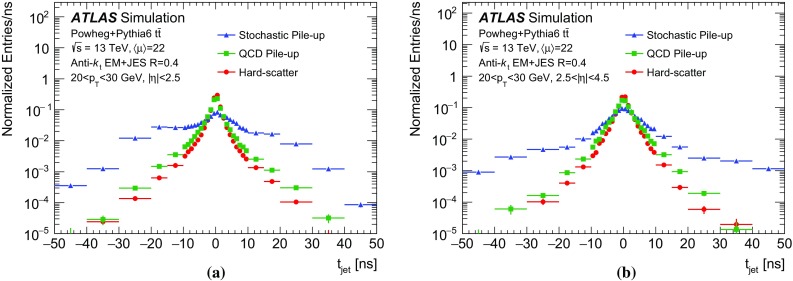
Distribution of the jet timing $t_\mathrm {jet}$ for hard-scatter, QCD pile-up and stochastic pile-up jets in the **a** central and **b** forward region

Given the evidence presented in Sect. 3 that out-of-time pile-up plays an important role for stochastic jets, a direct handle consists of the timing information associated with the jet. The jet timing $t_\mathrm {jet}$ is defined as the energy-weighted average of the timing of the constituent clusters. In turn, the cluster timing is defined as the energy-weighted average of the timing of the constituent calorimeter cells. The jet timing distribution, shown in Fig. 5, is symmetric and centred at $t_\mathrm {jet}=0$ for both the hard-scatter and pile-up jets. However, the significantly wider distribution for stochastic jets reveals the large out-of-time pile-up contribution. For jets with $20<p_{\text {T}} {}<30$  $\text {GeV}$, requiring $|t_\mathrm {jet}|<12$ ns ensures that 20% of stochastic pile-up jets are rejected while keeping 99% of hard-scatter jets. In the following, this is always applied as a baseline requirement when identifying stochastic pile-up jets.

Stochastic jets can be further suppressed using shape information. Being formed from a random collection of particles from different interactions, stochastic jets lack the characteristic dense energy core of jets originating from the showering and hadronization of a hard-scatter parton. The energy is instead spread rather uniformly within the jet cone. Therefore, pile-up mitigation techniques based on jet shapes have been shown to be effective in suppressing stochastic pile-up jets [2]. In this section, the challenges of this approach are presented, and different algorithms exploiting the jet shape information are described and characterized.

The jet width *w* is a variable that characterizes the energy spread within a jet. It is defined as

3 $$\begin{aligned} w = \frac{\sum _k{\Delta R (\mathrm {jet},k)p_{\mathrm {T}}^k}}{\sum _k{p_{\mathrm {T}}^k}}, \end{aligned}$$

where the index *k* runs over the jet constituents and $\Delta R (\mathrm {jet},k)$ is the angular distance between the jet constituent *k* and the jet axis. The jet width is a useful observable for identifying stochastic jets, as the average width is significantly larger for jets with a smaller fraction of energy originating from a single interaction.

In simulation the jet width can be computed using truth-particles (*truth-particle width*), as a reference point to benchmark the performance of the reconstructed observable. At detector level, the jet constituents are calorimeter topo-clusters. In general, topo-clustering compresses the calorimeter information while retaining its fine granularity. Ideally, each cluster captures the energy shower from a single incoming particle. However, the cluster multiplicity in jets decreases quickly in the forward region, to the point where jets are formed by a single cluster and the jet width can no longer be defined. An alternative approach consists of using as constituents the 11 by 11 grid of calorimeter towers in $\eta \times \phi$, centred around the jet axis. The use of calorimeter towers ensures a fixed multiplicity given by the $0.1\times 0.1$ granularity so that the jet width always contains jet shape information.

**Fig. 6:**
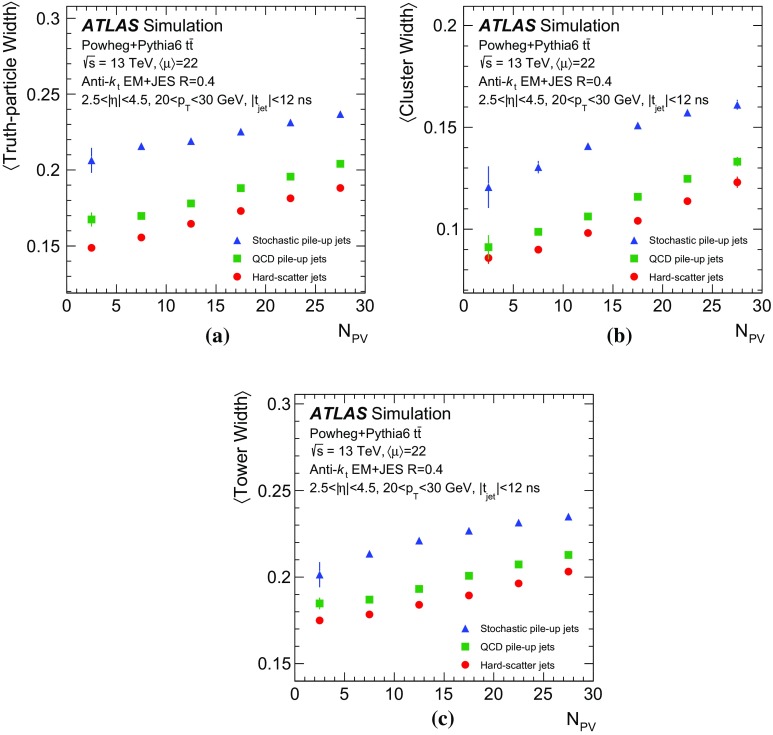
Dependence of the average jet width on the number of reconstructed primary vertices ($N_\mathrm {PV}$). The distributions are shown using **a** hard-scatter and in-time pile-up truth-particles, **b** clusters, or **c** towers as constituents

As shown in Fig. 6, the average jet width depends on the pile-up conditions. At higher pile-up values, a larger number of pile-up particles are likely to contribute to a jet, thus broadening the energy distribution within the jet itself. As a result, the width drifts towards higher values for hard-scatter, QCD pile-up, and stochastic jets. The difference in width between hard-scatter and QCD pile-up jets is due to the different underlying $p_{\text {T}}$ spectra. The spectrum of QCD pile-up jets is softer than that of the hard-scatter jets for the process considered (${t\bar{t}}$); therefore, a significant fraction of QCD pile-up jets are reconstructed with $p_{\text {T}}$ between 20 and 30 $\text {GeV}$because the stochastic and out-of-time component is larger than in hard-scatter jets.

**Fig. 7:**
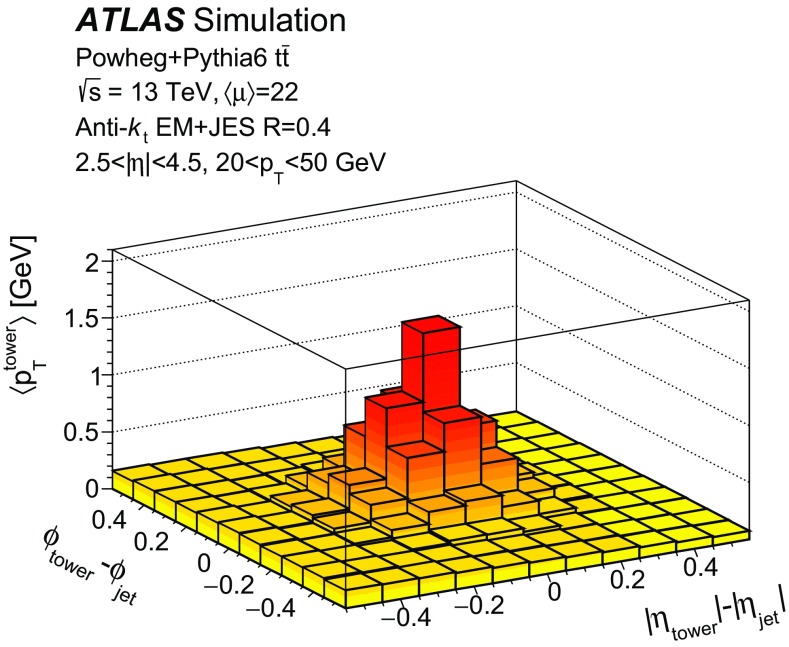
Distribution of the average tower $p_{\text {T}}$ for hard-scatter jets as a function of the angular distance from the jet axis in $\eta$ and $\phi$ in simulated ${t\bar{t}}$ events

Using calorimeter towers as constituents, it is possible to explore the $p_{\text {T}}$ distribution within a jet with a fixed $\eta \times \phi$ granularity. Figure 7 shows the two-dimensional $p_{\text {T}}$ distribution around the jet axis for hard-scatter jets. The distribution is symmetric in $\phi$, while the pile-up pedestal decreases with increasing $\eta$, as is expected in the forward region. A new variable, designed to exploit the full information about tower constituents, is considered. The two-dimensional^7^ $p_{\text {T}}$ distribution in the $\Delta \eta$–$\Delta \phi$ plane centred around the jet axis is fitted with a function

4 $$\begin{aligned} f = \alpha +\beta \Delta \eta +\gamma \mathrm {e}^{-\frac{1}{2}\left( \frac{\Delta \eta }{0.1}\right) ^2-\frac{1}{2}\left( \frac{\Delta \phi }{0.1}\right) ^2}. \end{aligned}$$

Both the width of the Gaussian component of the fit and the range in which the fit is performed are treated as jet-independent constants. The fit range, an $11\times 11$ tower grid, optimizes the balance between an improved constant ($\alpha$) and linear ($\beta$) term measurement by using a larger range and a decreased risk of including outside pile-up fluctuations by using a smaller range. On average, the jet tower $p_{\text {T}}$ distribution is symmetric with respect to $\Delta \phi$, and pile-up rejection at constant hard-scatter efficiency is improved by averaging the tower momenta at $|\Delta \phi |$ and $-|\Delta \phi |$ so that fluctuations are partially cancelled before performing the fit.

**Fig. 8:**
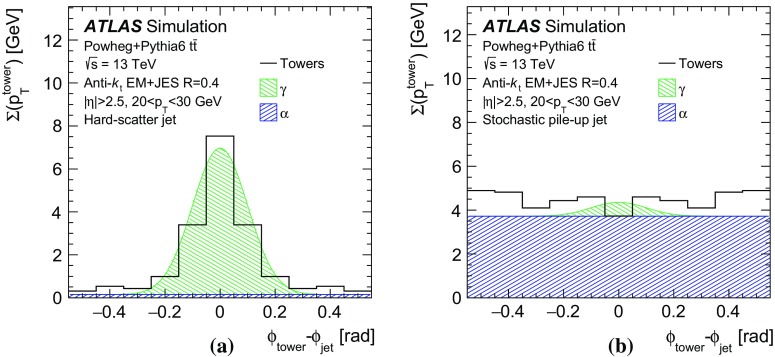
Symmetrized tower $p_{\text {T}}$ distribution projections in $\phi$ for an example **a** hard-scatter jet and **b** stochastic pile-up jet in simulated ${t\bar{t}}$ events. The *black histogram line* corresponds to the projection of the 2D tower distribution. The fit model closely follows the hard-scatter jet distribution, yielding a large Gaussian signal, while stochastic pile-up jets feature multiple smaller signals, away from the jet core

The constant ($\alpha$) and linear ($\beta$) terms in the fit capture the average stochastic pile-up contribution to the jet $p_{\text {T}}$ distribution, while the Gaussian term describes the $p_{\text {T}}$ distribution from the underlying hard-scatter or QCD pile-up jet. The parameter $\gamma$ therefore represents a stochastic pile-up-subtracted estimate of the $p_{\text {T}}$ of such a hard-scatter or QCD pile-up jet in a $\Delta R=0.1$ core assuming a Gaussian $p_{\text {T}}$ distribution of its constituent towers. By definition, $\gamma$ does not depend on the amount of pile-up in the event, but only on the stochastic nature of the jet.. In order to make the fitting procedure more robust, the Gaussian width parameter is fixed. While the width of a hard-scatter or QCD pile-up jet is expected to depend on the truth-particle jet $p_{\text {T}}$ and $\eta$, such dependence is negligible in the $p_{\text {T}}$ range relevant for these studies (20–50 $\text {GeV}$). Figure 8, showing projections of the tower distribution with the fit function overlaid, illustrates the characteristic peaking shape of pure hard-scatter jets compared with the flatter distribution in stochastic jets. The hard-scatter jet distribution displays the expected, sharply peaked distribution, while the stochastic pile-up jet distribution is flat with various off-centre features, reflecting the randomness of the underlying processes.

**Fig. 9:**
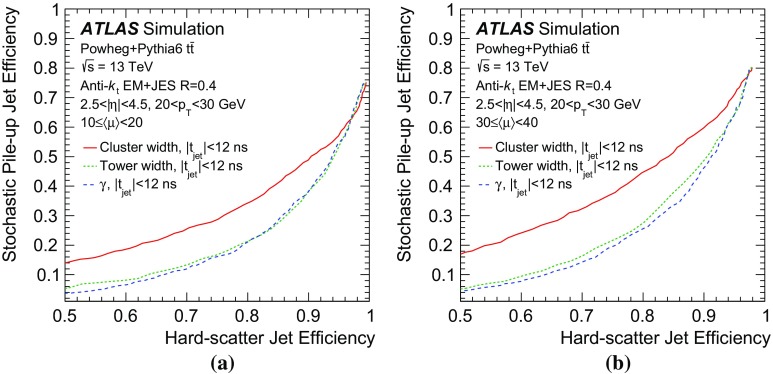
Efficiency for stochastic pile-up jets as a function of the efficiency for hard-scatter jets using different shape-based discriminants: **a** $10\le \langle \mu \rangle <20$ and **b** $30\le \langle \mu \rangle <40$ in simulated ${t\bar{t}}$ events

**Fig. 10:**
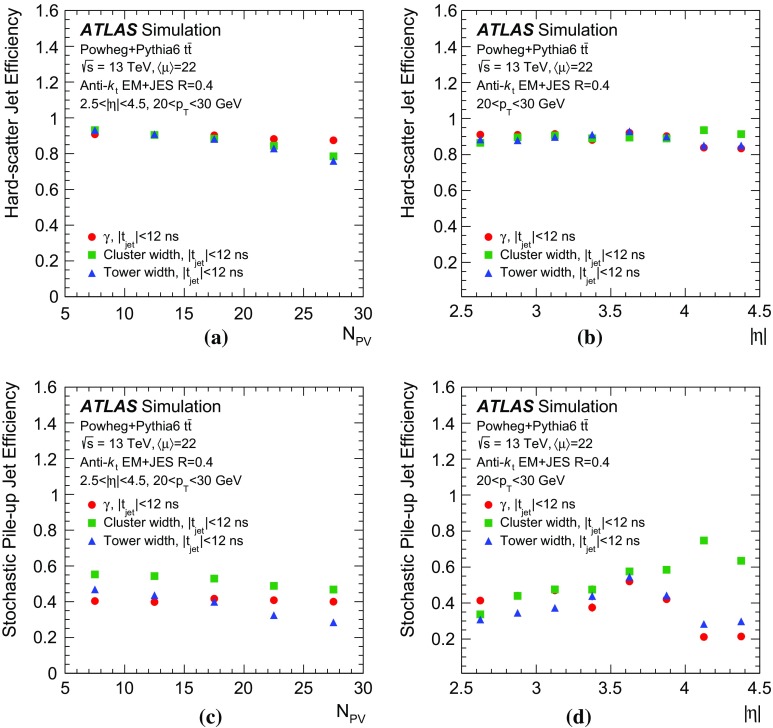
Hard-scatter jet efficiency as a function of **a** number of reconstructed primary vertices $N_\mathrm {PV}$ and **b** pseudorapidity $|\eta |$, as well as stochastic pile-up jet efficiency as a function of **c** number of reconstructed primary vertices $N_\mathrm {PV}$ and **d** pseudorapidity $|\eta |$ at 90% efficiency of selecting hard-scatter jets in simulated ${t\bar{t}}$ events

The performance of the $\gamma$ variable and of the cluster-based and tower-based widths is compared in Fig. 9, where the efficiency for stochastic pile-up jets is shown as a function of the hard-scatter jet efficiency. Each curve is obtained by applying an upper or lower bound on the jet width or $\gamma$, respectively, in order to select hard-scatter jets. The tower-based width outperforms the cluster-based width over the whole efficiency range, while the $\gamma$ variable performs similarly to the tower-based width. The hard-scatter efficiency and pile-up efficiency dependence on the number of reconstructed vertices in the event ($N_\mathrm {PV}$) and $\eta$ is shown in Fig. 10; the requirement for each discriminant is tuned so that an overall efficiency of 90% is achieved for hard-scatter jets. By construction, the performance of the $\gamma$ variable is less affected by the pile-up conditions than the two width variables.

The $\gamma$ parameter is a good discriminant for stochastic pile-up jets because it provides an estimate of the largest amount of $p_{\text {T}}$ in the jet originating from a single vertex. If there is no dominant contribution, the $p_{\text {T}}$ distribution does not feature a prominent core, and therefore $\gamma$ is close to zero. With this approach, all jets are effectively considered as QCD pile-up jets, and $\gamma$ is used to estimate their core $p_{\text {T}}$. Therefore, from this stage, the challenge of pile-up rejection is reduced to the identification and rejection of QCD pile-up jets, which is discussed in the following section.

## QCD pile-up jet tagging with topological information

While it has been shown that pile-up mitigation techniques based on jet shapes are effective in suppressing stochastic pile-up jets, such methods do not address QCD pile-up jets that are prevalent in the forward region. This section describes the development of an effective rejection method specifically targeting QCD pile-up jets.

QCD pile-up jets originate from a single $pp$ interaction where multiple jets can be produced. The total transverse momentum associated with each pile-up interaction is expected to be conserved;^8^ therefore all jets and central tracks associated with a given vertex can be exploited to identify QCD pile-up jets beyond the tracking coverage of the inner detector. The principle is clear if the dijet final state alone is considered. Forward pile-up jets are therefore identified by looking for a pile-up jet opposite in $\phi$ in the central region. The main limitation of this approach is that it only addresses dijet pile-up interactions in which both jets are reconstructed.

In order to address this challenge, a more comprehensive approach is adopted by considering the total transverse momentum of tracks and jets associated with each reconstructed vertex independently. The more general assumption is that the transverse momentum of each pile-up interaction should be balanced, and any imbalance would be due to a forward jet from one of the interactions.

In order to properly compute the transverse momentum of each interaction, only QCD pile-up jets should be considered. Consequently, the challenge of identifying forward QCD pile-up jets using transverse momentum conservation with central pile-up jets requires being able to discriminate between QCD and stochastic pile-up jets in the central region.

### A discriminant for central pile-up jet classification

Discrimination between stochastic and QCD pile-up jets in the central region can be achieved using track and vertex information. This section describes a new discriminant built for this purpose.

The underlying features of QCD and stochastic pile-up jets are different. Tracks matched to QCD pile-up jets mostly originate from a vertex PV$_i$ corresponding to a pile-up interaction ($i\ne 0$), thus yielding $R_\mathrm {pT} ^i>R_\mathrm {pT} ^0$ for a given jet. Such jets have large values of $R_\mathrm {pT} ^i$ with respect to the pile-up vertex *i* from which they originated. Tracks matched to stochastic pile-up jets are not likely to originate from the same interaction, thus yielding small $R_\mathrm {pT} ^i$ values with respect to any vertex *i*. This feature can be exploited to discriminate between these two categories. For stochastic pile-up jets, the largest $R_\mathrm {pT} ^i$ value is going to be of similar size as the average $R_\mathrm {pT} ^i$ value across all vertices, while a large difference will show for QCD jets, as most tracks originate from the same pile-up vertex.

**Fig. 11:**
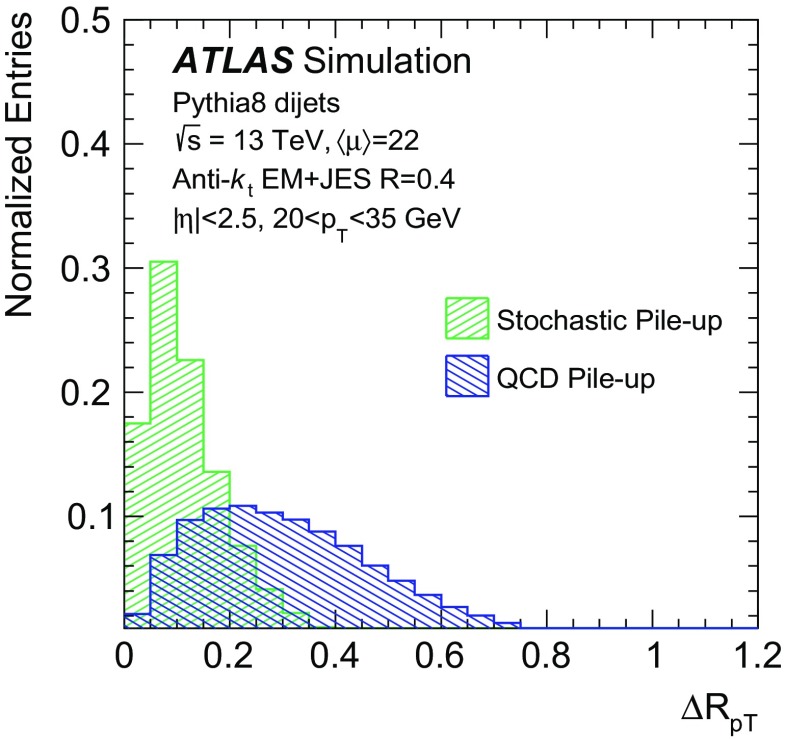
Distribution of $\Delta R_\mathrm {pT}$ for stochastic and QCD pile-up jets, as observed in dijet events with Pythia8.186 pile-up simulation

Thus, the difference between the leading and median values of $R_\mathrm {pT} ^i$ for a central jet, $\Delta R_\mathrm {pT}$, can be used for distinguishing QCD pile-up jets from stochastic pile-up jets in the central region, as shown in Fig. 11. A minimum $\Delta R_\mathrm {pT}$ requirement can effectively reject stochastic pile-up jets. In the following a $\Delta R_\mathrm {pT} >0.2$ requirement is applied for central jets with $p_{\text {T}} < 35 \,\text {GeV}$. Above this threshold the fraction of stochastic pile-up jets is negligible, and all pile-up jets are therefore assumed to be QCD pile-up jets irrespective of their $\Delta R_\mathrm {pT}$ value. The choice of threshold depends on the pile-up conditions. This choice is tuned to be optimal for the collisions considered in this study, with an average of 13.5 interactions per bunch crossing.

**Fig. 12:**
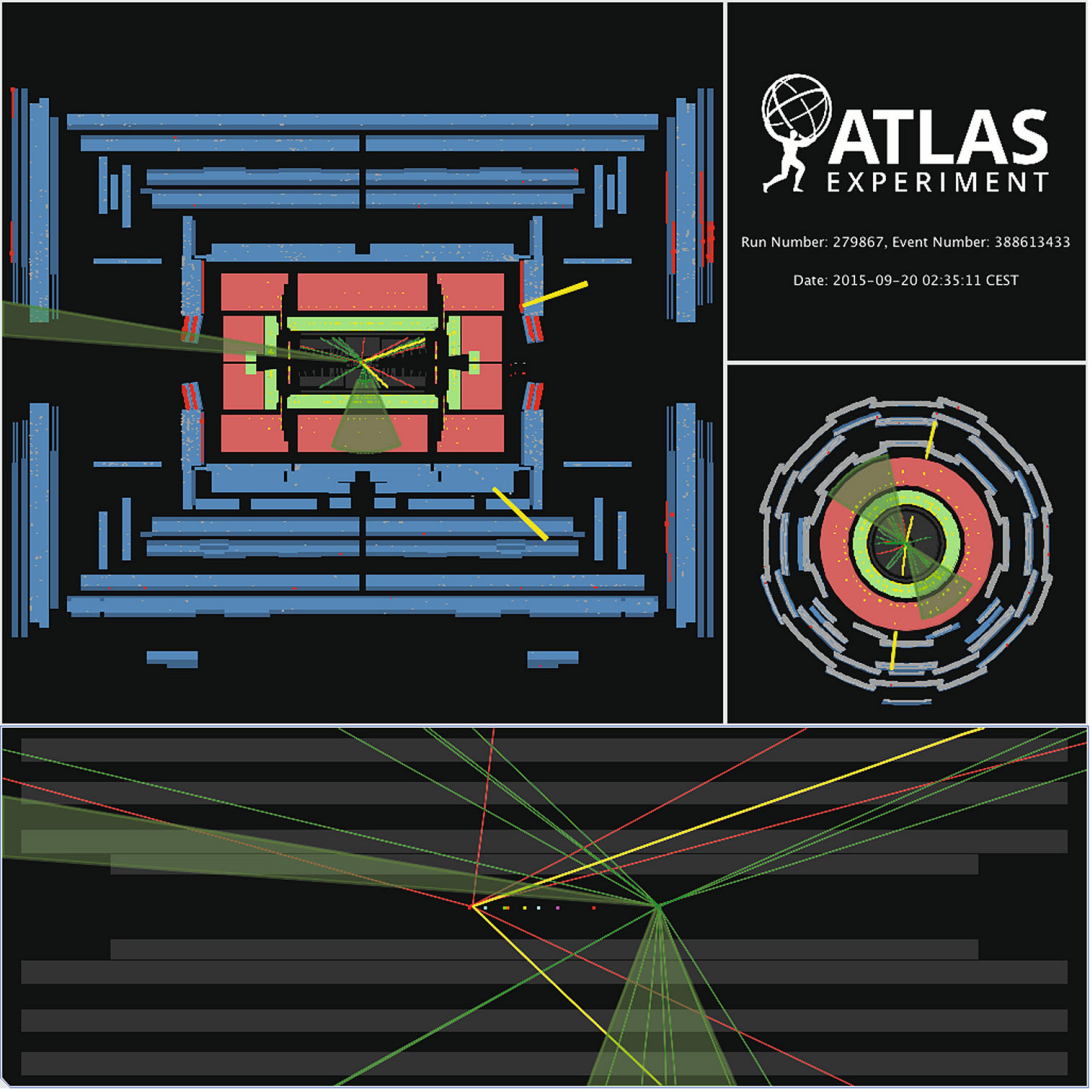
Display of candidate $Z (\rightarrow \mu \mu )$ event (muons in *yellow*) containing two QCD pile-up jets. Tracks from the primary vertex are in *red*, those from the pile-up vertex with the highest $\sum p_\mathrm {T}^2$ are in *green*. The *top panel* shows a transverse and longitudinal view of the detector, while the *bottom panel* shows the details of the event in the ID in the longitudinal view

The total transverse momentum of each vertex is thus computed by averaging, with a vectorial sum, the total transverse momentum of tracks and central jets assigned to the vertex. The jet–vertex matching is performed by considering the largest $R_\mathrm {pT} ^i$ for each jet. The transverse momentum vector ($\varvec{p}_\mathrm {T}$) of a given forward jet is then compared with the total transverse momentum of each vertex in the event. If there is at least one pile-up vertex in the event with a large total vertex transverse momentum back-to-back in $\phi$ with respect to the forward jet, the jet itself is likely to have originated from that vertex. Figure 12 shows an example event, where the $\varvec{p}_\mathrm {T}$ of a forward pile-up jet is back-to-back with respect to the total transverse momentum of the vertex from which it is expected to have originated.

### Forward jet vertex tagging algorithm

The procedure is referred to as *forward jet vertex tagging* (f$\mathrm {JVT}$). The main parameters for the forward JVT algorithm are thus the maximum JVT value, $\mathrm {JVT} _\mathrm {max}$, to reject central hard-scatter jets and the minimum $\Delta R_\mathrm {pT}$ requirement to ensure the selected pile-up jets are QCD pile-up jets. $\mathrm {JVT} _\mathrm {max}$ is set to 0.14 corresponding to an efficiency of selecting pile-up jets of 93% in dijet events. The minimum $\Delta R_\mathrm {pT}$ requirement defines the operating point in terms of efficiency for selecting QCD pile-up jet and contamination from stochastic pile-up jets. A minimum $\Delta R_\mathrm {pT}$ of 0.2 is required, corresponding to an efficiency of $70\%$ for QCD pile-up jets and $20\%$ for stochastic pile-up jets in dijet events. The selected jets are then assigned to the vertex PV$_i$ corresponding to the highest $R_\mathrm {pT} ^i$ value. For each pile-up vertex *i*, $i\ne 0$, the missing transverse momentum $\langle \varvec{p}_{\mathrm {T},i}^\mathrm {miss}\rangle$ is computed as the weighted vector sum of the jet ($\varvec{p}_\mathrm {T}^\mathrm {jet}$) and track ($\varvec{p}_\mathrm {T}^\mathrm {track}$) transverse momenta:

5 $$\begin{aligned} \langle \varvec{p}_{\mathrm {T},i}^\mathrm {miss}\rangle =-\frac{1}{2}\left( \sum _{\mathrm {tracks \in PV}_i}k\varvec{p}_\mathrm {T}^\mathrm {track} + \sum _{\mathrm {jets \in PV}_i}\varvec{p}_\mathrm {T}^\mathrm {jet}\right) . \end{aligned}$$

The factor *k* accounts for intrinsic differences between the jet and track terms. The track component does not include the contribution of neutral particles, while the jet component is not sensitive to soft emissions significantly below 20 $\text {GeV}$. The value $k=2.5$ is chosen as the one that optimizes the overall rejection of forward pile-up jets.

**Fig. 13:**
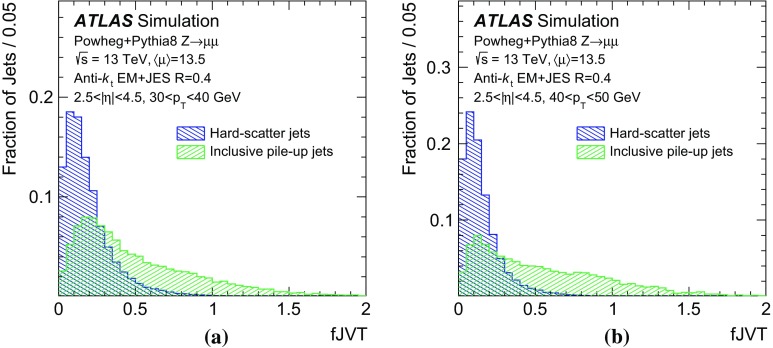
The $\mathrm {fJVT}$ distribution for hard-scatter (*blue*) and pile-up (*green*) forward jets in simulated $Z +$jets events with at least one forward jet with **a** $30<p_{\text {T}} <40$ $\text {GeV}$or **b** $40<p_{\text {T}} <50$ $\text {GeV}$

The $\mathrm {fJVT}$ discriminant for a given forward jet, with respect to the vertex *i*, is then defined as the normalized projection of the missing transverse momentum on $\varvec{p}_T^\mathrm {fj}$:

6 $$\begin{aligned} \mathrm {fJVT}_i = \frac{\langle \varvec{p}_{\mathrm {T},i}^\mathrm {miss}\rangle \cdot \varvec{p}_\mathrm {T}^\mathrm {fj}}{|\varvec{p}_\mathrm {T}^\mathrm {fj}|^2}, \end{aligned}$$

where $\varvec{p}_\mathrm {T}^\mathrm {fj}$ is the forward jet’s transverse momentum. The motivation for this definition is that the amount of missing transverse momentum in the direction of the forward jet needed for the jet to be tagged should be proportional to the jet’s transverse momentum. The forward jet is therefore tagged as pile-up if its $\mathrm {fJVT}$ value, defined as $\mathrm {fJVT} =\mathrm {max}_i(\mathrm {fJVT} _i)$, is above a threshold. The choice of threshold determines the pile-up rejection performance. The $\mathrm {fJVT}$ discriminant tends to have larger values for QCD pile-up jets, while the distribution for hard-scatter jets falls steeply, as shown in Fig. 13.

### Performance

**Fig. 14:**
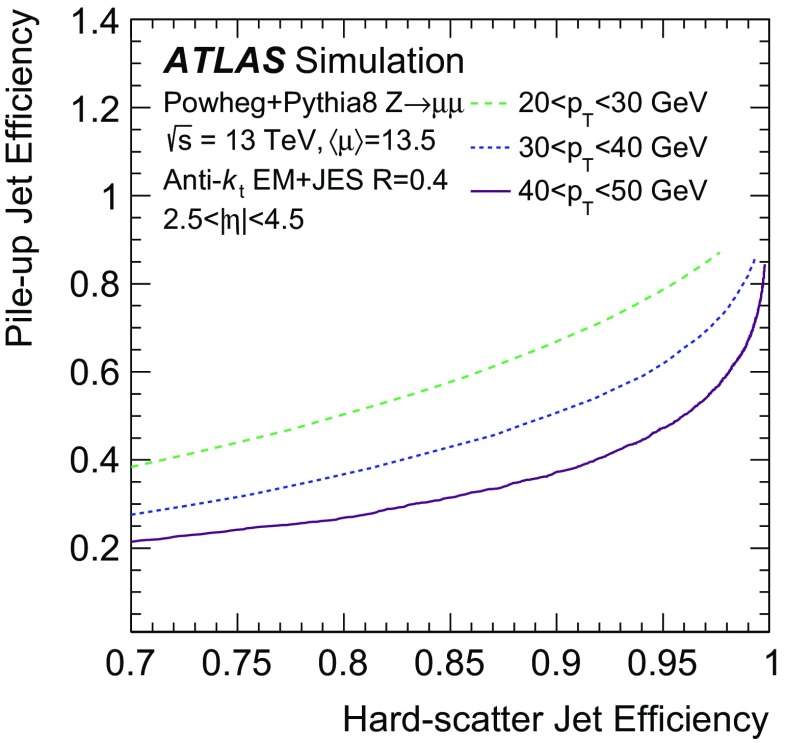
Efficiency for pile-up jets in simulated $Z +$jets events as a function of the efficiency for hard-scatter jets for different jet $p_{\text {T}}$ ranges.eps

Figure 14 shows the efficiency of selecting forward pile-up jets as a function of the efficiency of selecting forward hard-scatter jets when varying the maximum $\mathrm {fJVT}$ requirement.

**Fig. 15:**
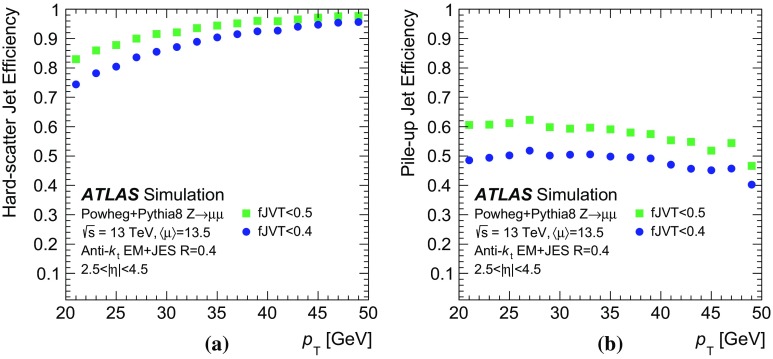
Efficiency for **a** hard-scatter jets and **b** pile-up jets as a function of the forward jet $p_{\text {T}}$ in simulated $Z +$jets events

**Fig. 16:**
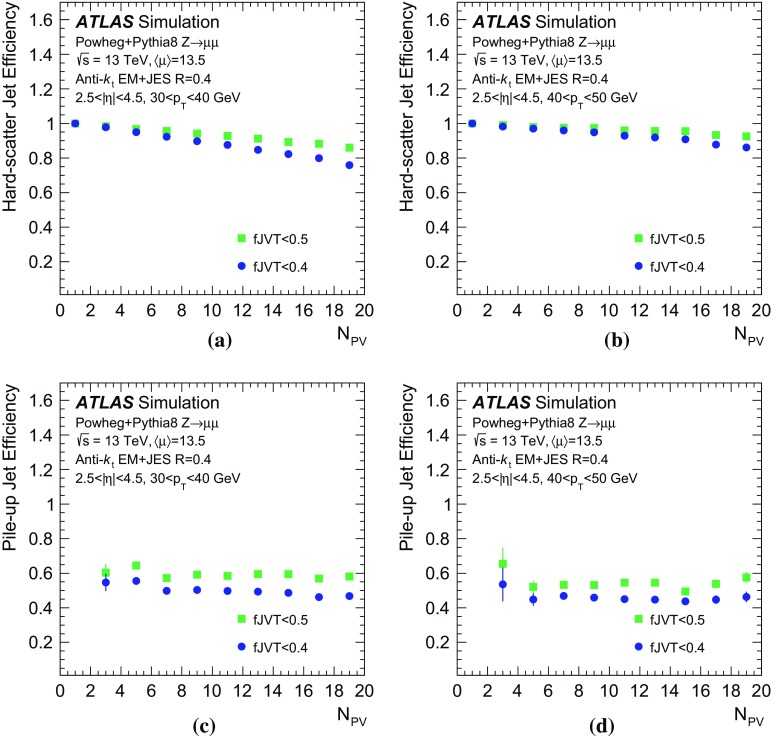
Efficiency in simulated $Z +$jets events as a function of $N_\mathrm {PV}$ for hard-scatter forward jets with **a** $30\,\text {GeV}<p_{\text {T}} <40\,\text {GeV}$ and **b** $40\,\text {GeV}<p_{\text {T}} <50\,\text {GeV}$, and for pile-up forward jets with **c** $30\,\text {GeV}<p_{\text {T}} <40\,\text {GeV}$ **d** $40\,\text {GeV}<p_{\text {T}} <50\,\text {GeV}$

Using a maximum $\mathrm {fJVT}$ of 0.5 and 0.4 respectively, hard-scatter efficiencies of 92 and 85% are achieved for pile-up efficiencies of 60 and 50%, considering jets with $20<p_{\text {T}} <50\,\text {GeV}$. The dependence of the hard-scatter and pile-up efficiencies on the forward jet $p_{\text {T}}$ is shown in Fig. 15. For low-$p_{\text {T}}$ forward jets, the probability of an upward fluctuation in the $\mathrm {fJVT}$ value is more likely, and therefore the efficiency for hard-scatter jets is slightly lower than for higher-$p_{\text {T}}$ jets. The hard-scatter efficiency depends on the number of pile-up interactions, as shown in Fig. 16, as busier pile-up conditions increase the chance of accidentally matching the hard-scatter jet to a pile-up vertex. The pile-up efficiency depends on the $p_{\text {T}}$ of the forward jets, due to the $p_{\text {T}}$-dependence of the relative numbers of QCD and stochastic pile-up jets.

### Efficiency measurements

The $\mathrm {fJVT}$ efficiency for hard-scatter jets is measured in $Z +\mathrm {jets}$ data events, exploiting a tag-and-probe procedure similar to that described in Ref. [1].

For $Z(\rightarrow \mu \mu )+$jets events, selected by single-muon triggers, two muons of opposite sign and $p_{\text {T}} >25\,\text {GeV}$ are required, such that their invariant mass lies between 66 and 116 $\text {GeV}$. Events are further required to satisfy event and jet quality criteria, and a veto on cosmic-ray muons.

Using the leading forward jet recoiling against the $Z$ boson as a probe, a signal region of forward hard-scatter jets is defined as the back-to-back region specified by $|\Delta \phi (Z, \mathrm {jet})| > 2.8$ rad. In order to select a sample pure in forward hard-scatter jets, events are required to have no central hard-scatter jets with $p_{\text {T}} >20 \,\text {GeV}$, identified with $\mathrm {JVT}$, and exactly one forward jet. The $Z$ boson is required to have $p_{\text {T}} > 20 \,\text {GeV}$, as events in which the $Z$ boson has $p_{\text {T}}$ less than the minimum defined jet $p_{\text {T}}$ have a lower hard-scatter purity. The above selection results in a forward hard-scatter signal region that is greater than 98% pure in hard-scatter jets relative to pile-up jets, as estimated in simulation.

**Fig. 17:**
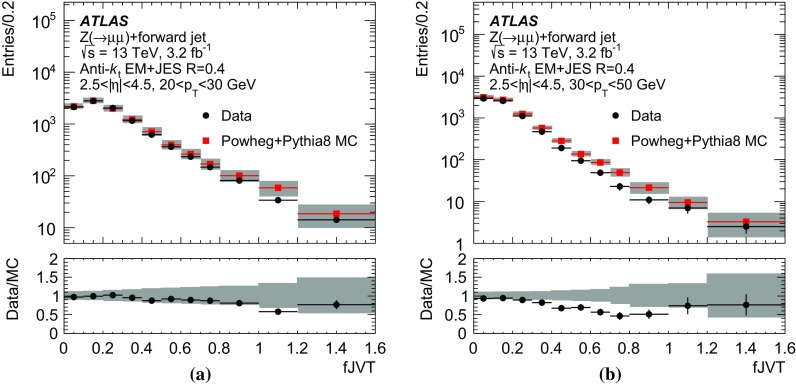
Distributions of $\mathrm {fJVT}$ for jets with $p_{\text {T}}$ **a** between 20 and 30  $\text {GeV}$and **b** between 30 and 50 $\text {GeV}$for data (*black circles*) and simulation (*red squares*). The *lower panels* display the ratio of the data to the simulation. The *grey bands* account for statistical and systematic uncertainties

The $\mathrm {fJVT}$ distributions for data and simulation in the signal region are compared in Fig. 17. The data distribution is observed to have fewer jets with high $\mathrm {fJVT}$ than predicted by simulation, consistent with an overestimation of the number of pile-up jets, as reported in Ref. [1].

The pile-up jet contamination in the signal region $N_{\mathrm {PU}}^\mathrm {signal}(|\Delta \phi (Z,\mathrm {jet}) |>2.8~\mathrm {rad})$ is estimated in a pile-up-enriched control region with $|\Delta \phi (Z,\mathrm {jet}) |<1.2$ rad, based on the assumption that the $|\Delta \phi (Z,\mathrm {jet}) |$ distribution is uniform for pile-up jets. The validity of such assumption was verified in simulation. The pile-up jet rate in data is therefore used to estimate the contamination of the signal region as

7 $$\begin{aligned} N_{\mathrm {PU}}^\mathrm {signal}(|\Delta \phi (Z,\mathrm {jet}) |>2.8~\mathrm {rad}) = \\ [N_\mathrm {j}^\mathrm {control}(|\Delta \phi (Z,\mathrm {jet}) |<1.2~\mathrm {rad}) - N_\mathrm {HS}(|\Delta \phi (Z,\mathrm {jet}) |<1.2~\mathrm {rad})] \cdot (\pi - 2.8~\mathrm {rad})/1.2~\mathrm {rad}, \end{aligned}$$

where ${N_\mathrm {j}^\mathrm {control}(|\Delta \phi (Z,\mathrm {jet}) |<1.2~\mathrm {rad})}$ is the number of jets in the data control region and ${N_\mathrm {HS}(|\Delta \phi (Z,\mathrm {jet}) |<1.2~\mathrm {rad})}$ is the expected number of hard-scatter jets in the control region, as predicted in simulation.

**Fig. 18:**
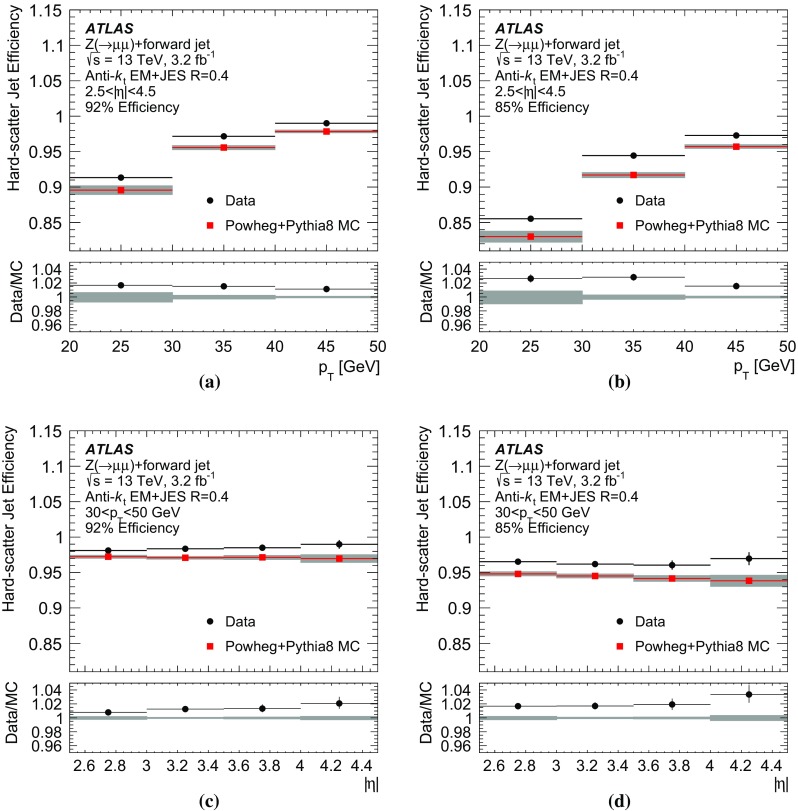
Efficiency for hard-scatter jets to pass $\mathrm {fJVT}$ requirements as a function of (**a**, **b**) $p_{\text {T}}$ and (**c**, **d**) $|\eta |$ for the (**a**, **c**) 92% ($\mathrm {fJVT} <0.5$) and (**b**, **d**) 85% ($\mathrm {fJVT} <0.4$) hard-scatter efficiency operating points of the $\mathrm {fJVT}$ discriminant in data (*black circles*) and simulation (*red squares*). The *lower panels* display the ratio of the data to the simulation. The *grey bands* account for statistical and systematic uncertainties

The hard-scatter efficiency is therefore measured in the signal region as

8 $$\begin{aligned} \varepsilon = \frac{N_\mathrm {j}^\mathrm {pass} - N_\mathrm {PU}^\mathrm {pass}}{N_\mathrm {j}^\mathrm {signal} - N_{\mathrm {PU}}^\mathrm {signal}}, \end{aligned}$$

where $N_\mathrm {j}^\mathrm {signal}$ and $N_\mathrm {j}^\mathrm {pass}$ denote respectively the overall number of jets in the signal region and the number of jets in the signal region satisfying the $\mathrm {fJVT}$ requirements. The terms $N_\mathrm {PU}^\mathrm {pass}$ and $N_\mathrm {PU}^\mathrm {signal}$ represent the overall number of pile-up jets in the signal region and the number of pile-up jets satisfying the $\mathrm {fJVT}$ requirements, respectively, and are both estimated from simulation. Figure 18 shows the hard-scatter efficiency evaluated in data and simulation. The uncertainties correspond to a 30% uncertainty in the number of pile-up jets and a 10% uncertainty in the number of hard-scatter jets in the signal region. The uncertainties are estimated by comparing data and simulation in the pile-up- and hard-scatter-enriched regions, respectively. The hard-scatter efficiency is found to be underestimated in simulation, consistent with the simulation overestimating the pile-up activity in data. The level of disagreement is observed to be larger at low jet $p_{\text {T}}$ and high $|\eta |$ and can be as large as about 3%. The efficiencies evaluated in this paper are used to define a calibration procedure accounting for this discrepancy. The uncertainties associated with the calibration and resolution of the jets used to compute $\mathrm {fJVT}$ are estimated in ATLAS analyses by recomputing $\mathrm {fJVT}$ for each variation reflecting a systematic uncertainty.

## Pile-up jet tagging with shape and topological information

The $\mathrm {fJVT}$ and $\gamma$ discriminants correspond to a twofold strategy for pile-up rejection targeting QCD and stochastic pile-up jets, respectively. However, as highlighted in Sect. 3, this classification is not well defined as all jets have a stochastic component. Therefore, it is useful to define a coherent strategy that addresses both the stochastic and QCD nature of pile-up jets at the same time.

The $\gamma$ parameter discussed in Sect. 4 provides an estimate of the $p_{\text {T}}$ in the core of the jet originating from the single interaction contributing the largest amount of transverse momentum to the jet. Therefore, the $\mathrm {fJVT}$ definition can be modified to exploit this estimation by replacing the jet $p_{\text {T}}$ with $\gamma$, so that

9 $$\begin{aligned} \mathrm {fJVT} _{\gamma } = \frac{\langle \varvec{p}_{\mathrm {T},i}^\mathrm {miss}\rangle \cdot \varvec{u}^\mathrm {fj}}{\gamma }, \end{aligned}$$

where $\varvec{u}^\mathrm {fj}$ is the unit vector representing the direction of the forward jet in the transverse plane.

**Fig. 19:**
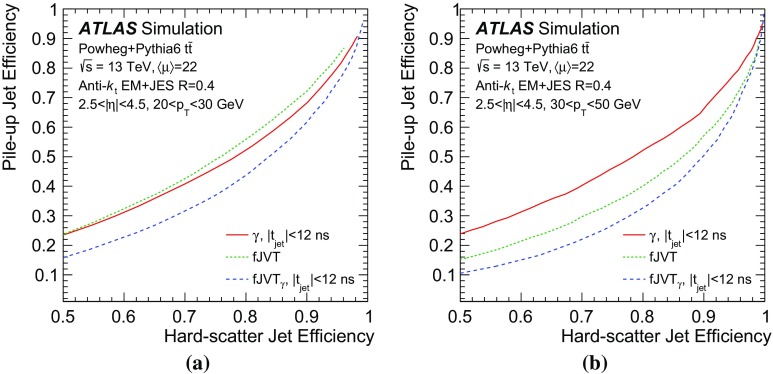
Efficiency for selecting pile-up jets as a function of the efficiency for selecting hard-scatter jets in simulated ${t\bar{t}}$ events for **a** jets with $20 \,\text {GeV}<p_{\text {T}} <30 \,\text {GeV}$ and **b** jets with $30\,\text {GeV}< p_{\text {T}} <50 \,\text {GeV}$

Figure 19 shows the performance of $\mathrm {fJVT} _{\gamma }$ compared with $\mathrm {fJVT}$ and $\gamma$ independently. The $\mathrm {fJVT} _{\gamma }$ discriminant outperforms the individual discriminants over the whole efficiency range. In samples enriched in QCD pile-up jets ($30<p_{\text {T}} < 50$ $\text {GeV}$), the $\mathrm {fJVT} _{\gamma }$ performance is driven by the topology information, while $\mathrm {fJVT} _{\gamma }$ benefits from the shape information for rejecting stochastic pile-up jets. A multivariate combination of $\mathrm {fJVT}$ and $\gamma$ discriminants was also studied and found to be similar in performance to $\mathrm {fJVT} _{\gamma }$.

## Impact on physics of Vector–Boson Fusion

In order to quantify the impact of forward pile-up rejection on a VBF analysis, the VBF $$H\rightarrow \tau \tau $$ signature is considered, in the case where the $$\tau $$ decays leptonically. The pile-up dependence of the signal purity (S/B) is studied in a simplified analysis in the dilepton channel. Several other channels are used in the analysis of VBF $$H\rightarrow \tau \tau $$ by ATLAS; the dilepton channel is chosen for this study by virtue of its simple selection and background composition. The dominant background in this channel originates from $$Z +$$jets production, where the $$Z $$ boson decays leptonically, either to electrons, muons, or a leptonically decaying $$\tau \tau $$ pair. The rate of $$Z $$ bosons produced in association with two jets satisfying the requirements targeting the VBF topology is extremely low. The requirements include large $$\Delta \eta $$ between the jets and large dijet invariant mass $$m_\mathrm {jj}$$. However, background events with forward pile-up jets often have large $$\Delta \eta $$ and $$m_\mathrm {jj}$$, mimicking the VBF topology. As a consequence, the background acceptance grows almost quadratically with the number of pile-up interactions. This section illustrates the mitigation of this effect that can be achieved with the pile-up rejection provided by $$\mathrm {fJVT} _{\gamma }$$.

The event selection used for this study was optimized using simulation without pile-up  [26]:

- The event must contain exactly two opposite-charge same-flavour leptons $\ell ^+\ell ^-$ (with $\ell =e$,$\mu$) with $p_{\text {T}}$ >15 $\text {GeV}$;
- The invariant mass of the lepton pair must satisfy $m_{\ell ^+\ell ^-}<66\,\text {GeV}$ or $m_{\ell ^+\ell ^-}>116\,\text {GeV}$;
- The magnitude of the missing transverse momentum must be larger than $40\,\text {GeV}$;
- The event must contain two jets with $p_{\text {T}} >20\,\text {GeV}$, one of which has $p_{\text {T}} >40\,\text {GeV}$. The absolute difference in rapidities $|\eta _{\mathrm {j}_1}-\eta _{\mathrm {j}_2}|$ must exceed 4.4 and the invariant mass of the two jets must exceed 700 $\text {GeV}$.
- For simulated VBF $H\rightarrow \tau \tau$ only, both jets are required to be truth-labelled as hard-scatter jets.

The impact of pile-up mitigation is emulated by randomly removing hard-scatter and pile-up jets to match the performance of a $\mathrm {fJVT} _{\gamma }$ requirement with 85% overall efficiency for hard-scatter jets with $20< p_{\text {T}} < 50~\text {GeV}$, as estimated in ${t\bar{t}}$ simulation with an average $\langle \mu \rangle$ of 13.5. The efficiencies are estimated as a function of the jet $p_{\text {T}}$ and the average number of interactions per bunch crossing.

**Fig. 20:**
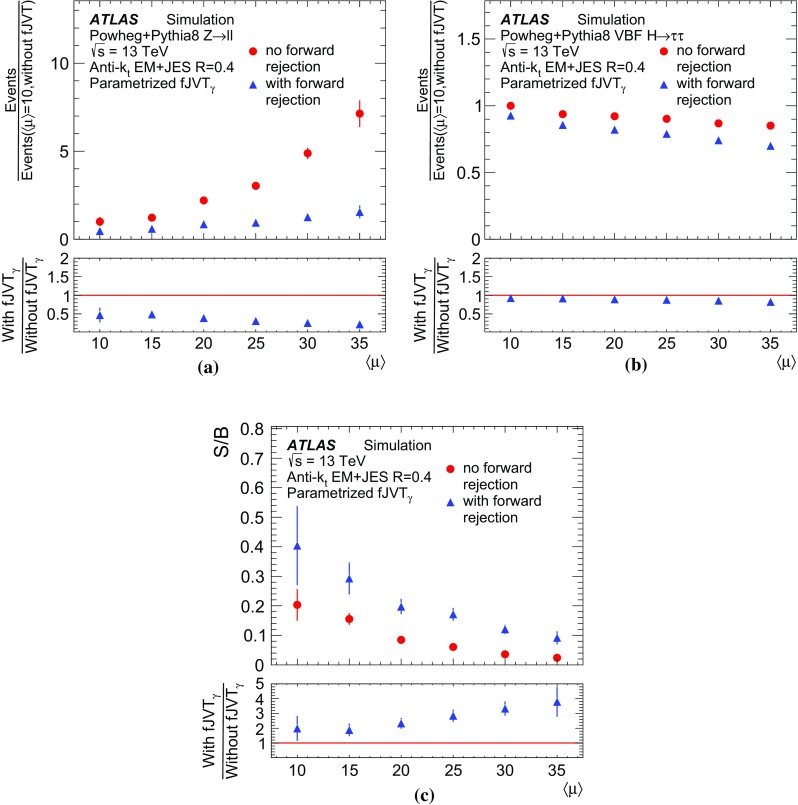
Relative expected yield variation of **a** $Z \rightarrow \ell \ell$ and **b** VBF $H\rightarrow \tau \tau$ events and **c** signal purity as a function of the number interactions per bunch crossing ($\langle \mu \rangle$), with different levels of pile-up rejection using $\mathrm {fJVT} _\gamma$. The expected signal and background yields at $\langle \mu \rangle =10$ are used as reference. Parameterized hard-scatter efficiency and pile-up efficiency are used. The *lower panels* display the ratio to the reference without pile-up rejection

Figure 20 shows the expected numbers of signal and background events, as well as the signal purity, as a function of $\langle \mu \rangle$. When going from $\langle \mu \rangle$ of 10 to 35, the expected number of background events grows by a factor of seven and the corresponding signal purity drops by a factor of eight, indicating that the presence of pile-up jets enhances the background acceptance. The slight decrease in signal acceptance is due to misidentification of pile-up jets as VBF jets. The $\mathrm {fJVT} _{\gamma }$ algorithm mitigates the background growth, at the expense of a signal loss proportional to the hard-scatter jet efficiency.^9^ Therefore, the degradation of the purity due to pile-up can be effectively reduced. For the specific final state and event selection under consideration, where $Z +$jets production is the dominant background, this results in about a fourfold improvement in signal purity at $\langle \mu \rangle =35$.

## Conclusions

The presence of multiple *pp* interactions per bunch crossing at the LHC, referred to as pile-up, results in the reconstruction of additional jets beside the ones from the hard-scatter interaction. The ATLAS baseline strategy for identifying and rejecting pile-up jets relies on matching tracks to jets to determine the *pp* interaction of origin. This strategy cannot be applied for jets beyond the tracking coverage of the inner detector. However, a broad spectrum of physics measurements at the LHC relies on the reconstruction of jets at high pseudorapidities. An example is the measurement of Higgs boson production through vector-boson fusion. The presence of pile-up jets at high pseudorapidities reduces the sensitivity for these signatures, by incorrectly reconstructing these final states in background events.

The techniques presented in this paper allow the identification and rejection of pile-up jets beyond the tracking coverage of the inner detector. The strategy to perform such a task is twofold. First, the information about the jet shape is used to estimate the leading contribution to the jet above the stochastic pile-up noise. Then the topological correlation among particles originating from a pile-up interaction is exploited to extrapolate the jet vertex tagger, using track and vertex information, beyond the tracking coverage of the inner detector to identify and reject pile-up jets at high pseudorapidities. When using both shape and topological information, approximately 57% of forward pile-up jets are rejected for a hard-scatter efficiency of about 85% at the pile-up conditions considered in this paper, with an average of 22 pile-up interactions. In events with 35 pile-up interactions, typical conditions for the LHC operations in the near future, 37, 48, and 51% of forward pile-up jets are rejected using, respectively, topological information, shape information, and their combination, for the same 85% hard-scatter efficiency.

A procedure is defined and used to measure the efficiency of identifying hard-scatter jets in 3.2 fb$^{-1}$of *pp* collisions at $\sqrt{s}=13\,\text {TeV}$ collected in 2015. The efficiencies are measured in data and estimated in simulation as a function of the jet kinematics. Discrepancies of up to approximately 3% are observed, mainly due to the modelling of pile-up events.

The impact of forward pile-up rejection algorithms presented here is estimated in a simplified study of Higgs boson production through vector-boson fusion and decaying into a $\tau \tau$ pair; the signal purity for the baseline selection under consideration, where $Z +$jets production is the dominant background, is enhanced by a factor of about four for events with 35 pile-up interactions.

## Footnotes

1
ATLAS uses a right-handed coordinate system with its origin at the nominal interaction point (IP) in the centre of the detector and the *z*-axis along the beam pipe. The *x*-axis points from the IP to the centre of the LHC ring, and the *y*-axis points upward. Cylindrical coordinates $(r, \phi )$ are used in the transverse plane, $\phi$ being the azimuthal angle around the beam pipe. The pseudorapidity is defined in terms of the polar angle $\theta$ as $\eta = - \ln \tan (\theta /2)$.

2 The jet reconstruction is described in Sect. 2.

3 The cell noise $\sigma _\mathrm {noise}$ is the sum in quadrature of the readout electronic noise and the cell noise due to pile-up, estimated in simulation [24, 25].

4 The EM scale corresponds to the energy deposited in the calorimeter by electromagnetically interacting particles without any correction accounting for the loss of signal for hadrons.

5 Truth particles are considered stable if their decay length $c \tau$ is greater than 1 cm. A truth particle is considered to be interacting if it is expected to deposit most of its energy in the calorimeters; muons and neutrinos are considered to be non-interacting.

6 The parameter *k* does not affect performance and is chosen to ensure that the $\mathrm {corrJVF}$ distribution stretches over the full range between 0 and 1.

7 The simultaneous fit of both dimensions was found to perform better than the fit of a 1D projection.

8 The cross-section of interactions producing high-$p_{\text {T}}$ neutrinos is negligible, compared to the rate of multijet events.

9 Most VBF events are characterized by one forward jet and one central jet.

## References

[1] ATLAS Collaboration, Performance of pile-up mitigation techniques for jets in $$pp$$ collisions at $$\sqrt{s}=8$$ TeV using the ATLAS detector. Eur. Phys. J. C **76**, 581 (2016). doi:10.1140/epjc/s10052-016-4395-z. arXiv:1510.03823 [hep-ex] 10.1140/epjc/s10052-016-4395-z PMC5335592 28316490

[2] CMS Collaboration, Pileup jet identification, CMS-PAS-JME-13-005 (2013). https://cds.cern.ch/record/1581583

[3] ATLAS Collaboration, The ATLAS experiment at the CERN Large Hadron Collider. JINST **3**, S08003 (2008). doi:10.1088/1748-0221/3/08/S08003

[4] ATLAS Collaboration, ATLAS Insertable B-layer technical design report, ATLAS-TDR-19 (2010). https://cds.cern.ch/record/1291633

[5] ATLAS Insertable B-Layer Technical Design Report Addendum, ATLAS-TDR-19-ADD-1 (2012). https://cds.cern.ch/record/1451888

[6] Abreu H (2010). Performance of the electronic readout of the ATLAS liquid argon calorimeters. JINST.

[7] ATLAS Collaboration, Measurement of the inelastic proton–proton cross-section at $$\sqrt{s}=7$$ TeV with the ATLAS detector. Nat. Commun. **2**, 463 (2011). doi:10.1038/ncomms1472. arXiv:1104.0326 [hep-ex] 10.1038/ncomms1472 PMC3220302 21897374

[8] T. Sjöstrand, S. Mrenna, P.Z. Skands, A brief introduction to PYTHIA 8.1. Comput. Phys. Commun. **178**, 852 (2008). doi:10.1016/j.cpc.2008.01.036. arXiv:0710.3820 [hep-ph]

[9] S. Carrazza, S. Forte, J. Rojo, Parton distributions and event generators (2013). arXiv:1311.5887 [hep-ph]

[10] ATLAS Collaboration, ATLAS Pythia 8 tunes to $$7\;{\rm TeV}$$ data, ATL-PHYS-PUB-2014-021 (2014). https://cds.cern.ch/record/1966419

[11] Alioli S, Nason P, Oleari C, Re E (2010). A general framework for implementing NLO calculations in shower Monte Carlo programs: the POWHEG BOX. JHEP.

[12] Frixione S, Nason P, Oleari C (2007). Matching NLO QCD computations with Parton Shower simulations: the POWHEG method. JHEP.

[13] Nason P (2004). A new method for combining NLO QCD with shower Monte Carlo algorithms. JHEP.

[14] Lai H-L, Guzzi M, Huston J, Li Z, Nadolsky PM (2010). New parton distributions for collider physics. Phys. Rev. D.

[15] T. Sjöstrand, S. Mrenna, P.Z. Skands, PYTHIA 6.4 physics and manual. JHEP **0605**, 026 (2006). doi:10.1088/1126-6708/2006/05/026. arXiv:hep-ph/0603175 [hep-ph]

[16] Skands PZ (2010). Tuning Monte Carlo generators: the Perugia tunes. Phys. Rev. D.

[17] Pumplin J, Stump D, Huston J, Lai H, Nadolsky PM (2002). New generation of parton distributions with uncertainties from global QCD analysis. JHEP.

[18] ATLAS Collaboration, Example ATLAS tunes of Pythia8, Pythia6 and Powheg to an observable sensitive to Z boson transverse momentum, ATL-PHYS-PUB-2013-017 (2013). https://cds.cern.ch/record/1629317

[19] Lange DJ (2001). The EvtGen particle decay simulation package. Nucl. Instrum. Methods A.

[20] ATLAS Collaboration, Summary of ATLAS Pythia 8 tunes, ATL-PHYS-PUB-2012-003 (2012). https://cds.cern.ch/record/1474107

[21] Martin A, Stirling W, Thorne R, Watt G (2009). Parton distributions for the LHC. Eur. Phys. J. C.

[22] ATLAS Collaboration, The ATLAS simulation infrastructure. Eur. Phys. J. C **70**, 823 (2010). doi:10.1140/epjc/s10052-010-1429-9. arXiv:1005.4568 [physics.ins-det]

[23] Agostinelli S (2003). GEANT4: a simulation toolkit. Nucl. Instrum. Methods A.

[24] ATLAS Collaboration, Topological cell clustering in the ATLAS calorimeters and its performance in LHC Run 1 (2016). arXiv:1603.02934 [hep-ex] 10.1140/epjc/s10052-017-5004-5 PMC5586976 28943797

[25] ATLAS Collaboration, Jet energy measurement with the ATLAS detector in proton-proton collisions at $$\sqrt{s}=7$$ TeV. Eur. Phys. J. C **73**, 2304 (2013). doi:10.1140/epjc/s10052-013-2304-2. arXiv:1112.6426 [hep-ex]

[26] ATLAS Collaboration, Expected performance of the ATLAS experiment- detector. Trigger Phys. (2009). arXiv:0901.0512 [hep-ex]

[27] ATLAS Collaboration, Early inner detector tracking performance in the 2015 data at $$\sqrt{s} = 13\;\text{TeV}$$ (2015). https://cds.cern.ch/record/2110140

[28] Cacciari M, Salam GP, Soyez G (2008). The catchment area of jets. JHEP.

[29] Cacciari M, Salam GP, Soyez G (2008). The anti-$$k_t$$ jet clustering algorithm. JHEP.

[30] Cacciari M, Salam GP, Soyez G (2012). FastJet user manual. Eur. Phys. J. C.

[31] ATLAS Collaboration, Jet global sequential corrections with the ATLAS detector in proton–proton collisions at $$\sqrt{s} = 8\;\text{ TeV }$$. ATLAS-CONF-2015-002 (2015). https://cds.cern.ch/record/2001682

[32] A. Hoecker et al., TMVA: toolkit for multivariate data analysis. **040** (2007). arXiv:physics/0703039

[33] ATLAS Collaboration, Electron reconstruction and identification efficiency measurements with the ATLAS detector using the 2011 LHC proton–proton collision data. Eur. Phys. J. C **74**, 2941 (2014). doi:10.1140/epjc/s10052-014-2941-0. arXiv:1404.2240 [hep-ex] 10.1140/epjc/s10052-014-2941-0 PMC4371047 25814900

[34] ATLAS Collaboration, Muon reconstruction performance of the ATLAS detector in proton–proton collision data at $$\sqrt{s} =13$$ TeV. Eur. Phys. J. C **76**, 292 (2016). doi:10.1140/epjc/s10052-016-4120-y. arXiv:1603.05598 [hep-ex] 10.1140/epjc/s10052-016-4120-y PMC5321258 28280436

[35] ATLAS Collaboration, Performance of algorithms that reconstruct missing transverse momentum in $$\sqrt{s}= 8$$ TeV proton–proton collisions in the ATLAS detector (2016). arXiv:1609.09324 [hep-ex] 10.1140/epjc/s10052-017-4780-2 PMC5409168 28515666

[36] Cacciari M, Salam GP (2008). Pileup subtraction using jet areas. Phys. Lett. B.

[37] ATLAS Collaboration, ATLAS computing acknowledgements 20162017, ATL-GEN-PUB-2016-002. https://cds.cern.ch/record/2202407

